# Crosstalk Between NDP52 and LUBAC in Innate Immune Responses, Cell Death, and Xenophagy

**DOI:** 10.3389/fimmu.2021.635475

**Published:** 2021-03-19

**Authors:** Hirohisa Miyashita, Daisuke Oikawa, Seigo Terawaki, Daijiro Kabata, Ayumi Shintani, Fuminori Tokunaga

**Affiliations:** ^1^ Department of Pathobiochemistry, Graduate School of Medicine, Osaka City University, Osaka, Japan; ^2^ Department of Medical Statistics, Graduate School of Medicine, Osaka City University, Osaka, Japan

**Keywords:** NDP52, ubiquitin, LUBAC, NF-κB, apoptosis, xenophagy

## Abstract

Nuclear dot protein 52 kDa (NDP52, also known as CALCOCO2) functions as a selective autophagy receptor. The linear ubiquitin chain assembly complex (LUBAC) specifically generates the N-terminal Met1-linked linear ubiquitin chain, and regulates innate immune responses, such as nuclear factor-κB (NF-κB), interferon (IFN) antiviral, and apoptotic pathways. Although NDP52 and LUBAC cooperatively regulate bacterial invasion-induced xenophagy, their functional crosstalk remains enigmatic. Here we show that NDP52 suppresses canonical NF-κB signaling through the broad specificity of ubiquitin-binding at the C-terminal UBZ domain. Upon TNF-α-stimulation, NDP52 associates with LUBAC through the HOIP subunit, but does not disturb its ubiquitin ligase activity, and has a modest suppressive effect on NF-κB activation by functioning as a component of TNF-α receptor signaling complex I. NDP52 also regulates the TNF-α-induced apoptotic pathway, but not doxorubicin-induced intrinsic apoptosis. A chemical inhibitor of LUBAC (HOIPIN-8) cancelled the increased activation of the NF-κB and IFN antiviral pathways, and enhanced apoptosis in *NDP52*-knockout and -knockdown HeLa cells. Upon *Salmonella*-infection, colocalization of *Salmonella*, LC3, and linear ubiquitin was detected in parental HeLa cells to induce xenophagy. Treatment with HOIPIN-8 disturbed the colocalization and facilitated *Salmonella* expansion. In contrast, HOIPIN-8 showed little effect on the colocalization of LC3 and *Salmonella* in *NDP52*-knockout cells, suggesting that NDP52 is a weak regulator in LUBAC-mediated xenophagy. These results indicate that the crosstalk between NDP52 and LUBAC regulates innate immune responses, apoptosis, and xenophagy.

## Introduction

Protein ubiquitination, a crucial post-translational modification, is catalyzed by ubiquitin-activating enzyme (E1), ubiquitin-conjugating enzyme (E2), and ubiquitin ligase (E3), and regulates numerous cellular functions, including proteasomal degradation, membrane trafficking, DNA repair, and signal transduction, by generating various types of ubiquitin linkages in the “ubiquitin code” (1, 2). Linear ubiquitin chain assembly complex (LUBAC) is composed of the HOIL-1L (also known as RBCK1), HOIP (RNF31), and SHARPIN subunits, and generates the N-terminal Met1-linked linear polyubiquitin chain (3–5). LUBAC is required for the regulation of the canonical nuclear factor-κB (NF-κB) activation pathway, interferon (IFN) regulatory factor 3 (IRF3)-mediated antiviral response, and cell death (5). Moreover, dysfunctions in LUBAC and linear ubiquitin-binding proteins, such as NF-κB-essential modulator (NEMO), optineurin (OPTN), and A20, are associated with various disorders (6–10). Indeed, we previously reported that OPTN selectively binds to linear ubiquitin through the UBAN domain, and plays a crucial role in the suppression of NF-κB activity (8). Furthermore, the amyotrophic lateral sclerosis-associated *OPTN* mutations, such as E478G and Q398X, abrogated the inhibitory effects on LUBAC-mediated NF-κB activation, and increased caspase activation (7, 8). These results suggested that LUBAC affects the physiological functions of OPTN.

To modulate the LUBAC activity, we developed α,β-unsaturated carbonyl-containing compounds, HOIPIN-1 (from HOIP-inhibitor-1) and its potent derivative HOIPIN-8 (11–13). HOIPINs are powerful and specific LUBAC inhibitors that suppress the LUBAC-mediated proinflammatory cytokine-induced NF-κB activation and pathogen-associated molecular patterns-induced IFN antiviral pathways, by modifying the active site Cys885 and thus specifically inhibiting the RING-HECT-hybrid reaction in HOIP (13). Indeed, we showed that HOIPINs suppressed the enhanced NF-κB activation in *OPTN*-deficient cells.

Nuclear dot protein 52 kDa (NDP52, also known as calcium binding and coiled-coil domain 2, CALCOCO2) was originally identified as an antigen protein localized in nuclear domain 10 (14, 15). NDP52, as well as OPTN, p62/SQSTM1, NBR1, and Tax1-binding protein 1 (TAX1BP1), functions as an autophagy cargo receptor that recognizes substrates for selective autophagy, including proteins, organelles, and pathogens, in a ubiquitin-dependent or independent manner, and links to ATG8/LC3 *via* LC3-interacting regions (LIRs) (16). In particular, NDP52 and OPTN are critical for the selective autophagy of damaged mitochondria (mitophagy) and invading microorganisms (xenophagy) (16–18). Bacteria that have invaded mammalian cells are initially restricted in vacuoles or phagosomes; however, some escape to the cytoplasm by disruption of the phagosomal or vacuolar membrane (19). NDP52 binds to galectin 8 (Gal8) (20), which recognizes bacterial carbohydrates in the cytoplasm and bridges to LC3 in autophagosomes. Moreover, ubiquitinated and ruptured phagosomal and bacterial membranes are recognized by NDP52 for autophagic degradation (21–23). Importantly, Noad and co-workers reported that LUBAC is recruited to the bacterial surface *via* HOIP, and linear ubiquitin is part of the ubiquitin coat of invading *Salmonella* (24). Subsequently, the recruited NEMO and OPTN, linear ubiquitin-binding UBAN domain-containing proteins, function in NF-κB and xenophagy, respectively. Furthermore, van Wijk et al. reported that OTULIN, a linear ubiquitin chain-specific deubiquitinase, plays a major role in the regulation of linear ubiquitin in the bacterial coat, which affects the recruitment of NEMO and the activation of canonical IKK (25). A more recent report showed that the linear ubiquitination of ATG13 by LUBAC and the deubiquitination by OTULIN are associated with autophagy initiation (26). Thus, NDP52 and linear ubiquitination are closely correlated in xenophagy; however, the detailed physiological crosstalk between NDP52 and LUBAC has remained elusive. In this study, we investigated the physiological roles of the crosstalk between NDP52 and LUBAC in innate immune responses, cell death, and xenophagy, using the LUBAC inhibitors, HOIPINs.

## Materials and Methods

### Reagents

The following reagents were obtained as indicated: zVAD-FMK (ZVAD) (ENZO Life Sciences), recombinant human TNF-α and IL-1β (BioLegend), poly(I:C) (HMW) (Invivogen), doxorubicin (Calbiochem), DAPI (Dojindo), blasticidin (Wako), pepstatin A, chloroquine, and cycloheximide (Sigma), E64d (Tokyo Chemical Industry), monoubiquitin, eight kinds of diubiquitins, linear (M1)-, K11-, K48-, and K63-tetraubiquitins (Boston Biochem), control siRNA (sc-37007) and *NDP52*-siRNA (sc-93738) (Santa Cruz Biotechnology), and BV6 (Genentech). HOIPIN-1 (2-[(1*E*)-3-(2-methoxyphenyl)-3-oxoprop-1-en-1-yl] benzoic acid sodium salt) and HOIPIN-8 (2-{(E)-3-[2,6-difluoro-4-(1H-pyrazol-4-yl)-phenyl]-3-oxo-propenyl}-4-(1-methyl-1H-pyraol-4-yl)-benzoic acid sodium salt) were prepared as described (11, 12).

### Plasmids

The open reading frames of cDNAs were amplified by reverse-transcription PCR. Mutants of these cDNAs were prepared by the QuikChange method, and the nucleotide sequences were verified. The cDNAs were ligated to the appropriate epitope sequences and cloned into the pcDNA3.1 (Invitrogen), pMAL-c2x (New England Biolabs), and pGEX6p-1 (Addgene) vectors. For lentiviral transduction, pCSII-CMV-RfA-IRES-Blast (RIKEN BioResource Research Center) was used.

### Cell Culture and Transfection

HeLa cells (ATCC), HEK293T cells (ATCC), A549 cells (ATCC), and *ATG7^-/-^* HeLa cells (27) (a generous gift from Prof. Yoshimori) were cultured in DMEM containing 10% fetal bovine serum (FBS) and antibiotics. Transfection experiments were performed using PEI (polyethylenimine) or lipofectamine RNAiMAX (Thermo Fisher). For the stable expression of the FLAG-His_6_-tagged NDP52-wild type (WT) or -D439R mutant in *NDP52* ^-/-^ HeLa cells, lentiviral infection followed by selection with 5 μg/ml blasticidin was performed.

### Luciferase Assay

HEK293T and A549 cells were cultured in 24-well plates, and co-transfected with the pGL4.32 [*luc2P*/NF-κB-RE/Hygro] vector (Promega) and the pRL-TK *Renilla* Luciferase control reporter vector (Promega). At 24 h after transfection by PEI, the cells were lysed and the luciferase activity was measured with a GloMax 20/20 luminometer (Promega), using the Dual-Luciferase Reporter Assay System (Promega). At 18 h after transfection, TNF-α (10 ng/ml) or IL-1β (1 ng/ml) was added to the medium. The cultures were incubated further for 6 h and then the cells were analyzed.

### Immunoprecipitation, SDS-PAGE, and Immunoblotting

Cells were lysed with 50 mM Tris-HCl, pH 7.5, 150 mM NaCl, 1% Triton X-100, 2 mM PMSF, and complete protease inhibitor cocktail (Sigma). Immunoprecipitation was performed using appropriate antibodies followed by Protein G agarose beads (GE Healthcare) at 4°C with gentle rotation. Immunoprecipitates were washed five times with the lysis solution. The samples were then separated by SDS-PAGE and transferred to PVDF membranes. After blocking in Tris-buffered saline containing 0.1% Tween-20 (TBS-T) with 5% skim-milk or bovine serum albumin (BSA), the membrane was incubated with the appropriate primary antibodies, diluted in TBS-T containing 5% w/v BSA, and then with horseradish peroxidase-conjugated secondary antibodies (GE Healthcare). The chemiluminescent images were obtained with an LAS4000 imaging analyzer (GE Healthcare) or a Fusion Solo S imaging system (Vilber).

### Antibodies

The following antibodies were used for immunoblot analyses: NDP52 (#9036; 1:1,000), P-IκBα (#9246; 1:1,000), IκBα (#4812; 1:1,000), P-p105 (#4806; 1:1,000), p105 (#3035; 1:1,000), P-p65 (#3033; 1:1,000), p65 (#8242; 1:1,000), P-IKKα/β (#2697; 1:1,000), P-IRF3 (#4947; 1:2,000), IRF3 (#4302; 1:1,000), P-TBK1 (#5483; 1:1000), TBK1 (#3504; 1:1000), caspase 8 (#4790; 1:1,000), cleaved caspase 8 (#9496; 1:1,000), caspase 3 (#9662; 1:1,000), cleaved caspase 3 (#9661; 1:1,000), caspase 9 (#9502; 1:1,000), Bid (#8762; 1:1,000), PARP (#9542; 1:1,000), and Atg7 (#8558; 1:1000) were obtained from Cell Signaling. HOIL-1L (sc-393754; 1:250), ubiquitin (P4D1) (sc-8017; 1:1,000), TNFR1 (sc-8436; 1:1,000), β-actin (sc-47778; 1:1,000), MAVS (sc-166583; 1:1,000), and IKKα/β (sc-7607; 1:1,000) were purchased from Santa Cruz Biotechnology. HOIP (ab125189; 1:1,000) and NEMO (ab178872; 1:3,000) were purchased from Abcam. RIP1 (BD Biosciences, 610458; 1:1,000), tubulin (Cedarlane, CLT9002; 1:3,000), SHARPIN (Proteintech, 14626-1-AP; 1:3,000), linear ubiquitin (Millipore, clone LUB9, MABS451; 1:1,000), HA (Roche, 11867423001; 1:1,000), Myc (MBL, HRP-Conjugate, M192-7; 1:20,000), and DYKDDDDK (Wako, 1E6, 015-22391; HRP-Conjugate; 1:20,000) were also used. For immunoprecipitation, c-Myc (Santa Cruz Biotechnology, sc-40; 1μg), FLAG (Sigma-Aldrich, clone M2, F1840; 1μg), FADD (Proteintech, 14906-1-AP; 2μg), NDP52 (Abcam, ab68588; 2 μg), and normal rabbit IgG (MBL, PM035; 2 μg) were used. For immunofluorescence analyses, LC3 (MBL, clone 4E12; 1:100), linear ubiquitin (Genentech, 1F11/3F5/Y102L; 5 μg/ml), and NDP52 (abcam, ab68588; 1:200) were used as primary antibodies, and then anti-mouse IgG Alexa Fluor 488, anti-human IgG Alexa Fluor 647, and anti-rabbit Alexa Fluor 647 (Thermo Fisher, goat polyclonal; 1:1000) were used as secondary antibodies, respectively.

### Recombinant Proteins

Expression vectors of maltose-binding protein (MBP)-fused wild type (WT)-NDP52 and the NDP52-D439R mutant, and MBP-LacZ were expressed in *Escherichia coli* Rosetta 2 (DE3) (Novagen) and purified using amylose resin (New England Biolabs). The glutathione *S*-transferase (GST)-fused NDP52-UBZ domain was expressed in *E. coli* BL21 (DE3)pLysS (Promega) and purified with a GSTrap column (GE Healthcare).

### Pulldown Assay

Linear polyubiquitin was prepared as described previously (28). Briefly, reaction mixture, containing 40 mM Tris-HCl, pH 7.5, 10 mM MgCl_2_, 0.6 mM dithiothreitol, 10 mM ATP, 1.4 mM ubiquitin (Boston Biochem), 1 μM baculovirus-expressed His-E1, 8 μM *E. coli*-expressed His-UbcH5c, and baculovirus-expressed His-HOIP/Myc-SHARPIN complex was incubated at 37°C for 3 h, and then heated at 60°C for 15 min. After centrifugation at 14,000 rpm for 30 min, the supernatant containing linear polyubiquitin was used. MBP-fusion proteins (0.8 μM) and tetraubiquitin (2 μM), diubiquitin (1 μM), monoubiquitin (1 μM), and linear polyubiquitin (1 μg) were incubated in reaction buffer (50 mM Tris-HCl, pH 7.5, 150 mM NaCl, 1 mM dithiothreitol, 0.1% NP-40 and 0.25 mg/ml BSA) at 37°C for 1 h, followed by the addition of amylose resin. Similarly, GST-fusion proteins were incubated with tetraubiquitin (3.4 μg), followed by glutathione Sepharose (GE Healthcare). The samples were further incubated at 4°C for 1 h with gentle rotation, and then the beads were washed three times with the reaction buffer without BSA, and analyzed by SDS–PAGE. The bound ubiquitin was detected by immunoblotting using an anti-ubiquitin antibody, and MBP- and GST-fusion proteins were stained with Coomassie Brilliant Blue (CBB).

For the pulldown assay with cell lysates, HEK293T cells were transiently transfected with plasmids encoding HA-tagged ubiquitin or its single-Lys mutants (K6, K11, K27, K29, K33, K48, and K63), and lysed with buffer containing 50 mM Tris-HCl, pH 7.5, 150 mM NaCl, 1 mM dithiothreitol, 0.1% NP-40, 2 mM PMSF, and protease inhibitor cocktails (Sigma). The lysates were incubated with MBP-fusion proteins in the reaction buffer for 1 h at 37°C, followed by the addition of amylose resin. The samples were further incubated at 4°C for 1 h with gentle rotation, and then the beads were washed three times with the reaction buffer without BSA.

For the pulldown assay with M1-TUBE (Tandem Ubiquitin Binding Entity), parental and *NDP52^-/–^-*HeLa cells (1×10^7^ cells) were stimulated with 1 μg/ml FLAG-tagged TNF-α, and lysed in 1 ml RIPA buffer, containing 50 mM Tris-HCl, pH 7.5, 150 mM NaCl, 1% NP-40, 0.5% sodium deoxycholate, 0.1% SDS, 2 mM EDTA, 2 mM PMSF, and protease inhibitor cocktails (Sigma), for 15 min on ice. The cell lysates were subjected to the pulldown with M1-TUBE Biotin (LifeSensors, UM306), and Dynabeads M-280 Streptavidin (Invitrogen) overnight at 4°C, and washed five times.

### Construction of Knockout HeLa Cells

The gRNA cloning vector and the pCAG-hCas9 vector were obtained from Addgene. The nucleotide sequence 5’-GAAGTTCTACATCCCTGGAGG-3’ in exon 2 of the human *NDP52* gene was selected as the target. These plasmids and a puromycin-resistant vector (pXS-Puro) were co-transfected into HeLa cells, and puromycin-resistant cell clones were selected by limiting dilution. Genome editing of the *NDP52* gene was screened by a BstNI digestion assay, and the mutations were confirmed by sequencing. The deficiency of the NDP52 protein was confirmed by immunoblotting.

For the knockout of the *RNF31* gene, which encodes HOIP, the nucleotide sequence 5’-TCAACCCTCAGGAAGCTCAGC-3’ in exon 2 of the human *RNF31* gene was selected as the target. Genome editing of the *RNF31* gene was screened by a BtsCI digestion assay, and the mutations were confirmed by sequencing. The deficiency of the HOIP protein was confirmed by immunoblotting.

### Quantitative PCR (qPCR)

Cell lysis, reverse-transcription, and qPCR were performed with a SuperPrep Cell Lysis RT Kit for qPCR (TOYOBO) and Power SYBR Green PCR Master Mix (Life Technologies), according to the manufacturers’ instructions. Quantitative real-time PCR was performed with a Step-One-Plus PCR system (Applied Biosystems) by the ΔΔCT method, using the following oligonucleotides: human *IL-6* sense, 5’-AGCCACTCACCTCTTC-3’, and human *IL-6* anti-sense, 5’-GCCTCTTTGCTGCTTT-3’; human *ICAM1* sense, 5’-GTGGTAGCAGCCGCAGT-3’, and human *ICAM1* anti-sense, 5’-TTCGGTTTCATGGGGGT-3’; human *TNF-α* sense, 5’-GCCGCATCGCCGTCTC-3’, and human *TNF-α* anti-sense, 5’-CCTCAGCCCCCTCTGG-3’; human *BIRC3* sense, 5’-AGATGAAAATGCAGAGTCATCAAT-3’, and human *BIRC3* anti-sense, 5’-CATGATTGCATCTTCTGAATGG-3’; human *IFIT2* sense, 5’-TGGTGGCAGAAGAGGAAGAT-3’, and human *IFIT2* anti-sense, 5’-GTAGGCTGCTCTCCAAGGAA-3’; human *ISG15* sense, 5’-GCGAACTCATCTTTGCCAGTA-3’, and human *ISG15* anti-sense, 5’-CCAGCATCTTCACCGTCAG-3’; and human *GAPDH* sense, 5’-AGCAACAGGGTGGTGGAC-3’, and human *GAPDH* anti-sense, 5’-GTGTGGTGGGGGACTGAG-3’.

### TNF Receptor Complex I and Complex II Analyses

The TNFR complex I analysis was performed as described previously (10). Briefly, parental, *NDP52^-/–^-*HeLa cells, and *HOIP^-/–^-*HeLa cells (2×10^7^ cells) were stimulated with 1 μg/ml FLAG-tagged TNF-α, and lysed in 1 ml lysis buffer, containing 50 mM Tris-HCl, pH 7.5, 150 mM NaCl, 1% Triton X-100, 2 mM PMSF, and protease inhibitor cocktails (Sigma), for 15 min on ice. The cell lysates were then immunoprecipitated with anti-FLAG M2 magnetic beads (Sigma) overnight at 4°C, and immunoprecipitates were washed five times with the lysis solution.

For the complex II analysis, parental and *NDP52^-/^* ^-^–HeLa cells (2×10^7^ cells) were stimulated with 20 μg/ml CHX, 20 ng/ml TNF-α, with or without 30 μM HOIPIN-8, and lysed in 1 ml lysis buffer, containing 50 mM Tris-HCl, pH 7.5, 150 mM NaCl, 1% Triton X-100, 2 mM PMSF, and protease inhibitor cocktails (Sigma), for 15 min on ice. The cell lysates were then immunoprecipitated using an anti-FADD antibody, followed by Protein G agarose beads (GE Healthcare) at 4°C for 2 h with gentle rotation. Subsequently, the samples were washed five times.

### Cell Survival Assay

The number of viable cells was measured with a Cell Counting Kit-F (DOJINDO), based on the degradation of Calcein-AM, and an ATP-based CellTiter-Glo Luminescent Cell Viability Assay (Promega). For quantifying cellular cytotoxicity, a trypan blue exclusion assay and a Cytotoxicity LDH Assay Kit-WST (DOJINDO), which is based on lactate dehydrogenase (LDH) release from damaged cells to the media, were used. Cell proliferation was measured with an xCELLigence RTCA S16 instrument (ACEA Biosciences, Inc.). For the experiments, the parental and *NDP52^-/–^-*HeLa cells were seeded in E-Plate VIEW 16 plates (ACEA Biosciences) at 20,000 cells/well, and monitored every 15 min for 24 h. The cells were then stimulated with TNF-α and CHX, and monitored every 15 min for 24 h. The data were analyzed with real-time cell analysis (RTCA) software and exported for statistical analysis.

### Xenophagy Assay

The xenophagy assay was basically performed as described (29) with minor modifications. Briefly, 1x10^5^ HeLa cells were seeded in 24-well plates, one day before infection. The cells were infected with *Salmonella enterica* serovar Typhimurium SR-11 χ3181 in penicillin/streptomycin-free cell culture medium, at a multiplicity of infection (MOI) of 100, for 15 min. The extracellular *Salmonella* cells were washed away with PBS, and then the residual bacteria were killed by culturing the cells in medium containing 50 μg/ml gentamicin for 40 min. The *Salmonella*-infected cells were cultured in medium with 10 μg/ml gentamicin, and lysed with extraction buffer (1% Triton X-100, 0.1% SDS in PBS) at the indicated times. The cell lysates were serially diluted with PBS, and small aliquots were spotted onto LB agar plates in quadruplicate. After 12 h incubation at 37°C, *Salmonella* colonies were counted manually to calculate the total number of bacteria in each well.

### Immunofluorescence Analysis

HeLa cells (5x10^4^) were seeded on collagen I-coated coverslips in 24-well plates, one day before infection. The cells were infected with mCherry-labeled *Salmonella* at a MOI of 300, and treated with gentamicin as described in the xenophagy assay protocol. The cells were fixed with phosphate buffered 4% paraformaldehyde at room temperature for 15 min, and then permeabilized/blocked in staining buffer (0.05% saponin, 10% FBS, 10 mM glycine in PBS) for 30 min. The cells were sequentially incubated with primary and secondary antibodies, diluted with the staining buffer, in a humidity box for 1 h. The stained cells were counterstained with DAPI and mounted onto glass slides with FluorSave (Millipore). The confocal fluorescence images of the prepared slides were captured with an LSM800 system (Carl Zeiss) using the following excitation laser, detection range and pinhole settings: DAPI (Laser: 405 nm, Detection range: 400-600 nm, Pinhole = 49 μm), Alexa488 (Laser: 488 nm, Detection range: 450-580 nm, Pinhole = 52 μm), mCherry (Laser: 561 nm, Detection range: 570-650 nm, Pinhole = 60 μm), and Alexa647 (Laser: 640 nm, Detection range: 645-700 nm, Pinhole = 66 μm). All images were acquired as 16-bit depth images with a 63X water-immersion objective lens by scanning each channel four separate times, at a speed of 3.18 μsec/pixel, for averaging. These images were analyzed with the accompanying ZEN software to depict the intensity profile plots and to calculate the mean fluorescence intensity of interest. *Pearson’s* and *Manders’* correlation coefficient between two independent channels were also determined using the ZEN software by thresholding with the signals obtained from negative control sample (non-infected cells stained with the secondary antibodies and DAPI only) as backgrounds. The captured images were processed with Fiji (ImageJ).

### Statistical Analysis

To examine the differences in the outcomes between the mutant groups, linear regression analyses were performed for each outcome variable separately. All pairwise comparisons were derived from the linear regression analyses. Furthermore, in order to compare the change of the normalized cell index over time among mutant groups, we conducted a multivariable non-linear regression analysis including a two-way interaction term between the indicator variable for the mutant groups and the time variable in addition to their main effect terms as explanatory variables. Moreover, the non-linear effect of the time variable on the outcome was considered using a restricted-cubic-spline with knot 5. In all regression models, we estimated the heteroskedasticity corrected standard errors and 95% confidence intervals using the Huber-White sandwich estimators for a robust variance-covariance matrix (30). Normality of the residuals of all regression models was assessed graphically. All residual plots appeared to show a good degree of normally or no meaningful skewness was detected. All p-values were adjusted for multiplicity using the Bonferroni method. All hypothesis tests were performed with a two-sided 5% significance level using R software (https://cran.r-project.org/).

## Results

### Ubiquitin-Binding Domain of NDP52 Is Crucial to Suppress Canonical NF-κB Signaling

**Figure 1:**
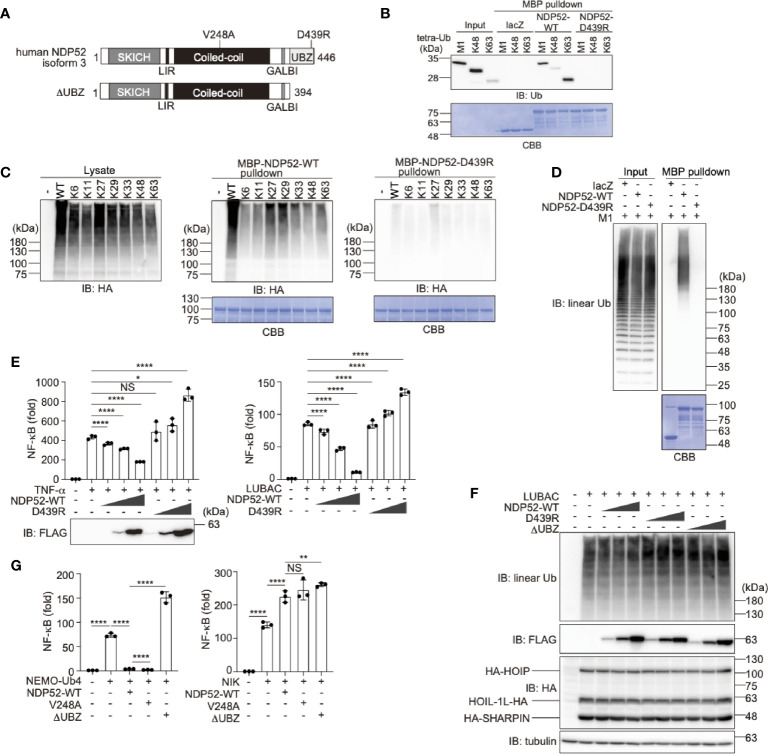
The ubiquitin-binding activity of NDP52 is indispensable for NF-κB suppression. **(A)** Domain structure of Wild-type (WT) human NDP52 isoform 3 and its ΔUBZ mutant. V248A is associated with Crohn’s disease (31), and the D439R substitution causes the defect in ubiquitin-binding (32). SKICH, SKIP carboxyl homology; LIR, LC3-interacting region; GALBI, galectin-8 binding; and UBZ, ubiquitin-binding zinc finger. **(B)** The UBZ domain of NDP52 functions as the ubiquitin-binding site. *In vitro* MBP pulldown experiments using linear (M1)-, K48-, or K63-linked tetraubiquitins and MBP-fused lacZ, NDP52-WT, and D439R mutant were performed, and the bound ubiquitin chain was detected by immunoblotting. The precipitated MBP-fusion proteins were visualized by Coomassie Brilliant Blue (CBB) staining. **(C)** Low ubiquitin selectivity of NDP52. HA-tagged single Lys mutants of ubiquitin were expressed in HEK293T cells, and the lysates were pulled down by MBP-NDP52-WT or -D439R mutant. The precipitated ubiquitin was detected by an anti-HA antibody, and the precipitated MBP-fusion proteins were detected by CBB staining. **(D)** Linear polyubiquitin-binding of NDP52. LUBAC-generated linear polyubiquitin was pulled down by the MBP-NDP52-WT or -D439R mutant *in vitro*. The precipitated ubiquitin was detected by an anti-linear ubiquitin antibody, and the precipitated MBP-fused proteins were detected by CBB staining. **(E)** NDP52-WT, but not the D439R mutant, suppresses NF-κB activity. Effects of increasing amounts (0.025 μg, 0.075 μg, and 0.25 μg/well) of WT and D439R mutant of FLAG-NDP52 were examined, in the presence of either 10 ng/ml TNF-α or the co-expression of LUBAC subunits in HEK293T cells, by the NF-κB luciferase assay. **(F)** NDP52 does not inhibit the linear ubiquitination activity of LUBAC. HA-tagged LUBAC subunits (1.0 μg HA-HOIP, 0.2 μg HOIL-1L-HA, and 0.2 μg HA-SHARPIN/well) and increasing amounts (0.1 μg, 0.3 μg, and 1.0 μg/well) of FLAG-NDP52-WT, -D439R, and -ΔUBZ were co-expressed in HEK293T cells, and cell lysates were immunoblotted with the indicated antibodies. **(G)** NDP52 suppresses the canonical NF-κB pathway. The C-terminally tetraubiquitin-fused NEMO (NEMO-Ub4), WT and mutants of NDP52, and/or NIK were co-transfected with the NF-κB luciferase reporter, as indicated, and luciferase activity was measured at 24 h-post transfection. **(E, G)** Data are shown as Means ± SD (*n* = 3) by Huber-White Sandwich estimators for variance-covariance structures corrected with Bonferroni method. *P < 0.05, **P < 0.01, ****P < 0.0001, NS, not significant.

Human NDP52 consists of the SKIP carboxyl homology (SKICH), LC3-interacting region (LIR), coiled-coil, galectin-8 binding (GALBI), and C-terminal ubiquitin-binding zinc finger (UBZ) domains (17, 19) (**Figure 1A**). The substitution of Asp439 by Arg (D439R) in the UBZ domain reportedly abolishes the ubiquitin-binding (31). Moreover, the genetic variant of Val248 to Ala (V248A) is associated with Crohn’s disease, a chronic inflammatory bowel disease (32). To confirm the ubiquitin-selectivity of NDP52, we initially performed pulldown experiments. Although MBP-fused NDP52-WT failed to pulldown either mono- or eight different kinds of diubiquitins (**Supplementary Figure 1A**), MBP-fused NDP52-WT, but not the D439R mutant, coprecipitated with linear (M1)-, K48-, or K63-tetraubiquitins with an affinity order of K63>M1>K48 (**Figure 1B**). The GST-fused UBZ domain (a.a. 394-446) of NDP52 also precipitated tetraubiquitins with a similar affinity order of K63>M1>K48>K11-tetraubiquitin (**Supplementary Figure 1B**). When we expressed the N-terminally HA-tagged single Lys mutants of ubiquitin in HEK293T cells, MBP-NDP52-WT, but not MBP-NDP52-D439R, bound all of the Lys-linked polyubiquitin chains, and seemed to have higher affinity toward K27- and K29-polyubiquitin chains, followed by K63-chain (**Figure 1C**). Since linear ubiquitination cannot be evaluated with an N-terminally tagged ubiquitin (3), we performed an *in vitro* MBP pulldown experiment using LUBAC-generated linear polyubiquitin (**Figure 1D**). The results revealed that MBP-NDP52-WT, but not the D439R mutant, coprecipitated long (>130 kDa) linear polyubiquitin chains. These results suggested that the UBZ domain of NDP-52 shows broad specificity to ubiquitin-linkages, including linear chains, although it may have higher affinity toward some atypical ubiquitin chains. Moreover, the mutation of Asp439 to Arg drastically abolishes the ubiquitin-binding of NDP52, including the M1-chain.

Next, to investigate the physiological functions of NDP52, we examined the effect of NDP52 overexpression on TNF-α- and LUBAC-mediated NF-κB activation, by a luciferase assay (**Figure 1E**). The increasing expression of NDP52-WT dose-dependently suppressed the NF-κB activation, whereas the overexpression of the D439R mutant failed to suppress, and rather increased, the NF-κB activity in HEK293T cells. Since the increasing expression of the WT-, or D439R- and ΔUBZ-mutants of NDP52 with LUBAC subunits showed no effect on the LUBAC-mediated linear ubiquitination (**Figure 1F**), NDP52 does not seem to directly inhibit the E3 activity of LUBAC. We previously showed that the C-terminally tetraubiquitin-fused NEMO (NEMO-Ub4), a mimic of linear ubiquitinated NEMO, fully activates the canonical NF-κB pathway (33). The NEMO-Ub4-induced NF-κB activation was suppressed by the WT and V248A variant of NDP52, but not by the ΔUBZ mutant (**Figure 1G**). In contrast, the noncanonical NF-κB activation pathway by NF-κB-inducing kinase (NIK) was not suppressed by the WT and mutants of NDP52. We further examined the effects of the overexpression of NDP52-WT, and V248A, D439R, and ΔUBZ mutants on the basal NF-κB activity. The increased expression of NDP52-WT and V248A had no effect on the basal NF-κB activity in HEK293T cells, whereas the overexpression of the D439R and ΔUBZ mutants dose-dependently enhanced the basal NF-κB activity (**Supplementary Figure 2A**). Similar effects of the NDP52-WT and D439R mutant were detected by a luciferase assay in A549 cells (**Supplementary Figure 2B**). These results suggested that the overexpression of ubiquitin-binding-defective mutants of NDP52 may affect the basal NF-κB activity. The V248A variant in *NDP52* is associated with Crohn’s disease, and thus the increased pathogen-induced NF-κB activation may contribute to the pathogenesis (31, 34). However, there were no differences in the canonical and noncanonical NF-κB activations with the V248A variant and NDP52-WT, suggesting that a different cellular phenomenon underlies this disease.

### NDP52 Binds NF-κB Signaling Factors, Including LUBAC

At present, 269 unique interactors and 409 raw interactions of NDP52 (CALCOCO2) are listed on the BioGRID site (https://thebiogrid.org/115535/summary/homo-sapiens/calcoco2.html). Intriguingly, the interaction of HOIL-1L (RBCK1) with NDP52 can be characterized by affinity capture mass spectrometry (35). Moreover, NF-κB signaling factors, such as TANK-binding kinase 1 (TBK1) (36), TBK1-binding protein 1 (TBKBP1) (36), MAVS (37), c-IAP2 (BIRC3) (38), IκB kinase (IKK)β (IKBKB) (39), IKKϵ (IKBKE) (21), NEMO (IKBKG) (40), TAB3 (21), A20 (TNFAIP3) (41), TRAF2 (42), TRAF3 (35), TRAF4 (43), and TRAF6 (40) are included as physiological interactors of NDP52. At present, NDP52 is reportedly involved in the TNF-α-induced NF-κB signal transduction pathway through binding with a ubiquitin editing complex, such as A20 (21), but it may play a more important role in the regulation of the NF-κB signaling pathway through binding with multiple factors, including LUBAC.

**Figure 2:**
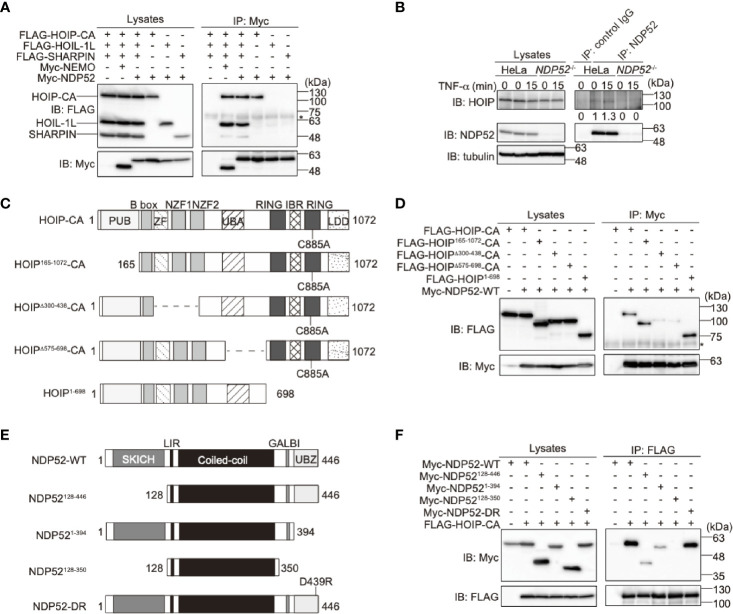
NDP52 binds LUBAC through HOIP. **(A)** HOIP binds NDP52. The FLAG-tagged HOIP C885A mutant (HOIP-CA), HOIL-1L, SHARPIN, and Myc-tagged NEMO or NDP52 were co-expressed in HEK293T cells, as indicated. The cell lysates and anti-Myc immunoprecipitates were immunoblotted with the indicated antibodies. *: nonspecific signal. **(B)** NDP52 physiologically interacts with LUBAC. Parental and *NDP52* ^-/–^-HeLa cells were stimulated with 1 μg/ml FLAG-TNF-α, and cell lysates and anti-NDP52 immunoprecipitates were subjected to immunoblotting with the indicated antibodies. Taking the intensity in control IgG immunoprecipitates as the background and the anti-NDP52 immunoprecipitates of HeLa without TNF-α stimulation as 1.0, the relative intensities of HOIP are shown. **(C)** Domain structure and mutants of HOIP. PUB: peptide:N-glycanase/UBA or UBX-containing proteins; ZF: zinc finger; NZF: Npl4-type zinc finger; UBA: ubiquitin-associated; RING: really interesting new gene; IBR: in-between RING; and LDD: linear ubiquitin determining. **(D)** The zinc fingers and UBA-flanking regions of HOIP are crucial for NDP52-binding. The full length and various mutants of FLAG-tagged HOIP were co-expressed with Myc-NDP52-WT in HEK293T cells, and immunoprecipitations followed by immunoblotting analyses were performed as indicated. *: nonspecific signal. **(E)** Domain structure and mutants of NDP52. **(F)** The SKICH and UBZ domains of NDP52 bind HOIP. A similar analysis to that in **(D)** was performed, using the WT and various mutants of Myc-NDP52 and FLAG-HOIP-CA.

To examine the binding of NDP52 with LUBAC, we performed a co-immunoprecipitation followed by an immunoblotting analysis in HEK293T cells (**Figure 2A**). To avoid the linear ubiquitin-mediated interaction, we used the active site mutant (C885A) of HOIP with HOIL-1L and SHARPIN. As a result, NDP52, as well as NEMO, a known substrate of LUBAC (44), efficiently coprecipitated with three components of the LUBAC complex. Among the LUBAC subunits, NDP52 bound HOIP alone, but not HOIL-1L or SHARPIN. The endogenous association of NDP52 and HOIP was also detected in HeLa cells (**Figure 2B**). The deletions of the zinc finger domains (a.a. 300-438) and UBA-flanking region (a.a. 575-698) of HOIP reduced NDP52-binding (**Figures 2C, D**). Furthermore, the deletion of either SKICH (a.a. 1-127) or the UBZ domain (a.a. 395-446) of NDP52 disturbed the HOIP-binding, whereas the coiled-coil region alone (a.a. 128-350) showed no affinity for HOIP (**Figures 2E, F**). These results indicated that NDP52 principally associates with LUBAC through HOIP, by biphasic interactions through the SKICH and UBZ domains.

To further confirm the binding of NDP52 with NF-κB signaling factors, we performed a co-immunoprecipitation assay using the WT and ΔUBZ mutant of NDP52 in HEK293T cells (**Supplementary Figure 3**). NDP52-WT efficiently associated with the reported NF-κB signaling factors, such as IKKβ, NEMO, IKKα, TRAF6, and A20. The deletion of the UBZ domain drastically reduced the association with NEMO, TRAF6, and A20. In contrast, IKKβ and IKKα substantially bound the ΔUBZ mutant of NDP52. Therefore, these different binding profiles of the WT- and ΔUBZ mutant of NDP52 with NF-κB signaling factors may correlate with the enhanced NF-κB activity by the overexpression of ubiquitin-binding defective mutants of NDP52.

### NDP52 Is a Negative Regulator of TNF-α and Antiviral Signaling pathways

**Figure 3:**
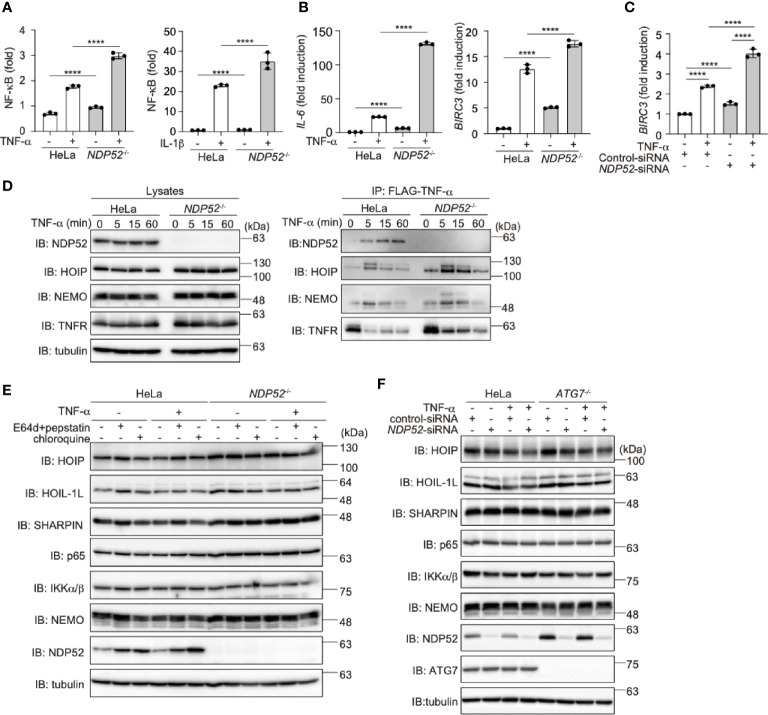
Genetic ablation of *NDP52* enhances canonical NF-κB activation. **(A)** Enhanced NF-κB activation in *NDP52* ^-/–^-HeLa cells. The NF-κB luciferase reporter was transfected into parental and *NDP52* ^-/–^-HeLa cells. The cells were then treated with or without 10 ng/ml TNF-α and 1 ng/ml IL-1β for 6 h, and the luciferase activity was measured. **(B)** Enhanced expression of NF-κB target genes in TNF-α-treated *NDP52* ^-/–^-HeLa cells. Parental- and *NDP52* ^-/–^-HeLa cells were stimulated with 10 ng/ml TNF-α for 1 h, and qPCR analyses of *IL-6* and *BIRC3* were performed. **(C)** Enhanced expression of *BIRC3* in *NDP52*-silenced HeLa cells. HeLa cells were transfected with control- or *NDP52*-siRNA and stimulated with 10 ng/ml TNF-α for 1 h, and then qPCR analysis was performed. **(A–C)** Data are shown as Means ± SD (*n* = 3) by Huber-White Sandwich estimators for variance-covariance structures corrected with Bonferroni method. ****P < 0.0001. **(D)** NDP52 is a component of TNFR complex I. Parental and *NDP52* ^-/–^-HeLa cells were stimulated with 1 μg/ml FLAG-TNF-α for the indicated periods, and cell lysates and anti-FLAG immunoprecipitates were subjected to immunoblotting with the indicated antibodies. **(E)** Lysosomal inhibitors do not affect the protein levels of LUBAC subunits or NF-κB signaling factors. Parental and *NDP52^-/–^*-HeLa cells were treated with E64d (10 μg/ml) and pepstatin A (10 μg/ml), or chloroquine (50 μM) for 8 h. Cell lysates were immunoblotted with the indicated antibodies. **(F)** *ATG7*-deficiency showed no effect on the turnover of NF-κB signaling factors. Parental and *ATG7^-/–^*-HeLa cells were transfected with control- or *NDP52*-siRNA and stimulated with 20 ng/ml TNF-α for 8 h. Cell lysates were immunoblotted by the indicated antibodies.

To further clarify the physiological functions of NDP52, we constructed *NDP52*-knockout (*NDP52* ^-/-^) HeLa cells by the CRIPSR/Cas9 method (**Supplementary Figure 4A**). The siRNA-mediated knockdown of *NDP52* had no effect on the background immunoreactive bands in the parental and *NDP52* ^-/–^-HeLa cells, suggesting the specific ablation of *NDP52* (**Supplementary Figure 4B**), Upon stimulation with TNF-α or IL-1β, the NF-κB luciferase activity was significantly up-regulated in *NDP52* ^-/–^-HeLa cells as compared to the parental cells (**Figure 3A**). Moreover, the expression of NF-κB target genes, such as *IL-6* and *BIRC3* (c-IAP2), was enhanced in TNF-α- and IL-1β-treated *NDP52* ^-/–^-HeLa cells (**Figure 3B**, **Supplementary Figure 4C**). We restored the NDP52-WT and D439R mutant into *NDP52* ^-/–^-HeLa cells, with comparable expression to that of the parental cells (**Supplementary Figure 4D**). Although the enhanced *IL-6* expression in *NDP52* ^-/-^ cells was significantly suppressed in NDP52-WT-restored cells, the restoration of the D439R mutant had minimal suppressive effects after TNF-α- and IL-1β-stimulation (**Supplementary Figures 4E, F**). In addition to *NDP52* ^-/–^-HeLa cells, the TNF-α-induced expression of *BIRC3* was upregulated in *NDP52*-siRNA-transfected HeLa cells, as compared to the control-siRNA-transfected cells (**Figure 3C**). These results suggested the inhibitory effect of NDP52 on the canonical NF-κB activation pathway.

Upon TNF-α stimulation, multiple proteins, such as receptor-interacting serine/threonine-protein kinase 1 (RIP1), IKK complex, and LUBAC, are recruited to the TNF receptor (TNFR) to form signaling complex I, which is crucial for TNF-α-mediated canonical NF-κB activation (45, 46). Importantly, we identified that NDP52 was recruited upon the FLAG-TNF-α treatment of the parental HeLa cells, but not *NDP52^-/–^*cells, clearly indicating that NDP52 is a component of TNFR signaling complex I (**Figure 3D**). Furthermore, the recruitment of NDP52 to TNFR complex I was partially suppressed in *HOIP^-/–^*cells (**Supplementary Figure 4G**), suggesting that the linear ubiquitination by LUBAC affects the association of NDP52 with TNFR upon TNF-α stimulation. Since the M1-TUBE pulldowns of linear ubiquitin from TNF-α-stimulated parental and *NDP52^-/–^*-HeLa cells showed similar levels of intracellular linear ubiquitin, NDP52 does not seem to inhibit the E3 activity of LUBAC (**Supplementary Figure 4H**).

To further examine whether NDP52 mediates the autophagic degradation of LUBAC and NF-κB signaling factors, we treated parental and *NDP52* ^-/–^-HeLa cells with lysosomal inhibitors, such as E64d+pepstatin or chloroquine (**Figure 3E**). However, these inhibitors had no effect on the protein levels of LUBAC subunits and NF-κB signaling factors in either the presence or absence of TNF-α stimulation. Moreover, the *NDP52* knockdown in *ATG7^-/-^*-HeLa cells had minimal effects on the intracellular amounts of LUBAC subunits and NF-κB signaling factors, in either the presence or absence of TNF-α stimulation (**Figure 3F**). Collectively, these results indicated that autophagy-induced lysosomal degradation is not involved in NDP52-mediated NF-κB regulation.

**Figure 4:**
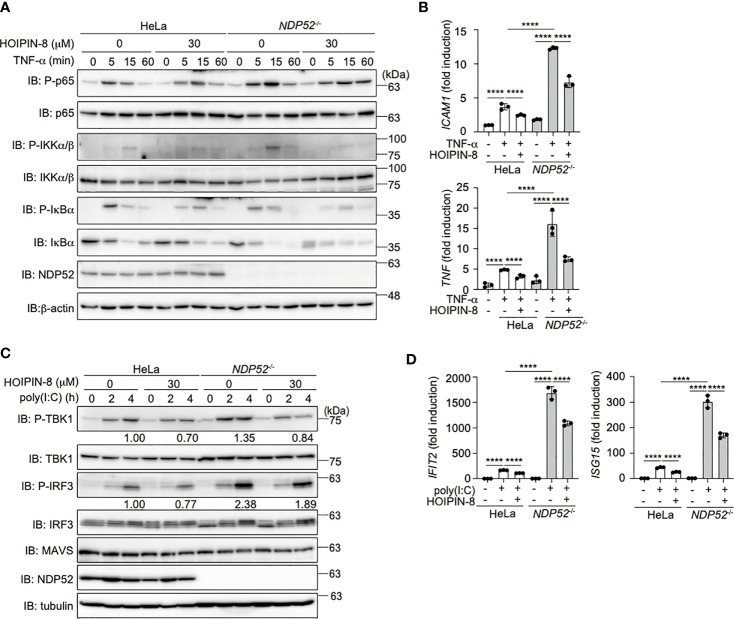
HOIPIN-8 suppresses the enhanced NF-κB and IFN antiviral signaling pathways in *NDP52* ^-/-^ cells. **(A)** HOIPIN-8 cancels the enhanced TNF-α signaling in *NDP52* ^-/–^-HeLa cells. Parental and *NDP52* ^-/–^-HeLa cells were stimulated with 10 ng/ml TNF-α for the indicated periods, in the absence or presence of 30 μM HOIPIN-8. Cell lysates were immunoblotted with the indicated antibodies. **(B)** The enhanced expression of NF-κB target genes in TNF-α-treated *NDP52* ^-/–^-HeLa cells is suppressed by HOIPIN-8. Parental and *NDP52* ^-/–^-HeLa cells were stimulated with TNF-α as in **(A)** for 1 h, and qPCR analyses of *ICAM1* and *TNF* were performed. **(C)** Poly (I:C)-mediated type I IFN signaling is enhanced in *NDP52* ^-/–^-HeLa cells. Parental and *NDP52* ^-/–^-HeLa cells were stimulated with 10 μg/ml poly(I:C) in either the presence or absence of 30 μM HOIPIN-8 for the indicated periods, and cell lysates were subjected to immunoblotting with the indicated antibodies. Taking the intensities of P-TBK1 and P-IRF3 in 4 h poly (I:C)-treated HeLa cells without HOIPIN-8 as 1.0, the relative intensities are shown. **(D)** Enhanced expression of IRF3 targets in *NDP52* ^-/–^-HeLa cells. The mRNA levels of *IFIT2* and *ISG15* in parental and *NDP52* ^-/–^-HeLa cells, treated as indicated for 4 h, were examined by qPCR. **(B, D)** Data are shown as Means ± SD (*n* = 3) by Huber-White Sandwich estimators for variance-covariance structures corrected with Bonferroni method. ****P < 0.0001.

To investigate the crosstalk between NDP52 and LUBAC, we treated parental and *NDP52^-/–^*-HeLa cells with LUBAC inhibitors, HOIPINs. The phosphorylation of p105, p65, and IKK, hallmarks of NF-κB activation, was upregulated in *NDP52^-/–^*-HeLa cell lysates as compared to the parental cells. However, the enhanced phosphorylation of NF-κB factors was dose-dependently cancelled in the presence of HOIPIN-8 or HOIPIN-1 (**Figure 4A**, **Supplementary Figure 5**). Moreover, the enhanced expression of NF-κB targets, such as *ICAM1* and *TNF*-α, in *NDP52^-/–^*-HeLa cells was suppressed in the presence of HOIPIN-8 (**Figure 4B**), suggesting that LUBAC inhibitors negated the increased NF-κB activation in *NDP52^-/–^*-HeLa cells.

When we transfected poly(I:C), a mimic of viral dsRNA, to activate the RIG-I-like receptors-mediated antiviral response, the phosphorylation of TBK1 and IRF3 was enhanced in *NDP52^-/–^*-cells, as compared to that in parental cells (**Figure 4C**), indicating the augmented activation of the IFN antiviral pathway by the genetic ablation of *NDP52*. However, we could not detect the degradation of TBK1, MAVS, and IRF3 after a 4 h-treatment with poly(I:C). In the presence of HOIPIN-8, the phosphorylation of TBK1 and IRF3 in parental and *NDP52^-/–^*-HeLa cells was partially suppressed. Furthermore, the expression of IRF3-target genes, such as *IFIT2* and *ISG15*, was upregulated in *NDP52^-/–^*-HeLa cells and suppressed by HOIPIN-8 (**Figure 4D**). These results indicated that NDP52 has a physiologically inhibitory effect on innate immune responses, such as the canonical NF-κB and type I IFN antiviral pathways.

### NDP52 Regulates TNF-α-Induced Apoptosis

**Figure 5:**
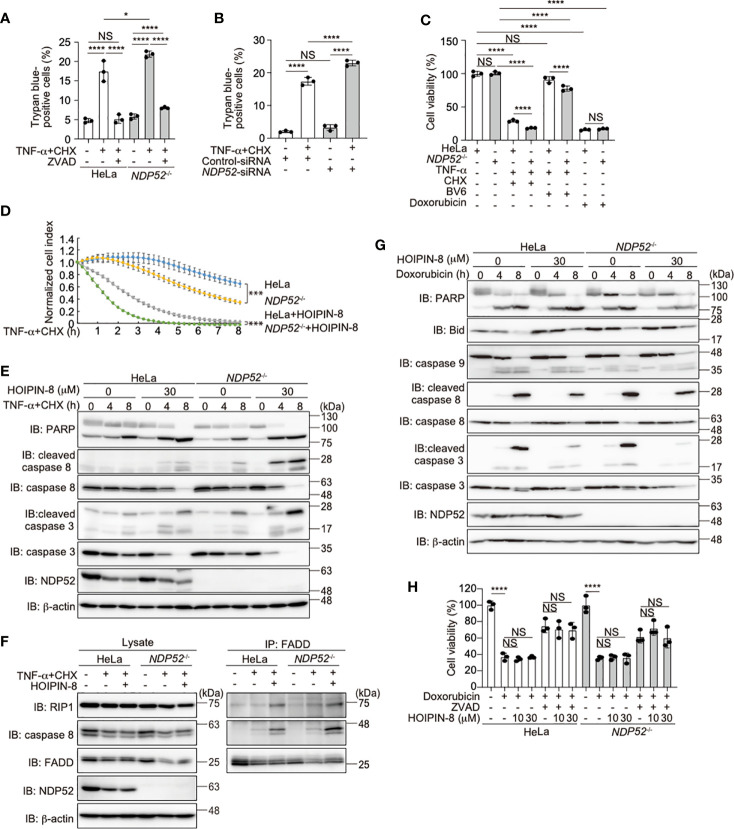
NDP52 suppresses the TNF-α-induced apoptosis. **(A)** TNF-α+CHX-induced cell death is enhanced in *NDP52* ^-/–^-HeLa cells. Parental and *NDP52* ^-/–^-HeLa cells were treated with 10 ng/ml TNF-α, 10 μg/ml CHX, and/or 20 μM ZVAD for 12 h, as indicated, and trypan-blue positive cells were counted. **(B)** Increased TNF-α-induced cell death in *NDP52*-silenced cells. Control- or *NDP52*-siRNA was transfected into HeLa cells, which were then treated with 10 ng/ml TNF-α+10 μg/ml CHX as indicated. Trypan-blue positive cells were counted as in **(A)**. **(C)** Reduced viability of *NDP52* ^-/-^ cells. Parental and *NDP52* ^-/–^-HeLa cells were treated with 10 ng/ml TNF-α, 10 μg/ml CHX, 4 μM BV6, and/or 25 μM doxorubicin as indicated for 24 h. The cell viability was then analyzed by an ATP-based assay. **(D)** HOIPIN-8 accelerated cell death. Parental and *NDP52* ^-/–^-HeLa cells were treated with 10 ng/ml TNF-α, 10 μg/ml CHX, and/or 30 μM HOIPIN-8, and analyzed by an xCELLigence real-time cell analyzer. Data are shown as mean ± s.e.m. (*n* = 4), and *P*-values for the comparison of the change of the normalized cell index over time between parental and *NDP52* ^-/–^-HeLa cells in the absence and presence of HOIPIN-8, respectively. ***P < 0.001. **(E)** Enhanced cleavages of caspases and PARP in TNF-α+CHX- and HOIPIN-8-treated *NDP52* ^-/–^-HeLa cells. Parental and *NDP52* ^-/–^-HeLa cells were treated with 10 ng/ml TNF-α, 10 μg/ml CHX, and/or 30 μM HOIPIN-8 as indicated, and cell lysates were analyzed by immunoblotting with the indicated antibodies. **(F)** Increased TNFR complex II formation in *NDP52* ^-/-^ cells. Parental and *NDP52* ^-/–^-HeLa cells were treated with 20 ng/ml TNF-α, 20 μg/ml CHX, and/or 30 μM HOIPIN-8 as indicated for 2.5 h, and cell lysates and anti-FADD immunoprecipitates were immunoblotted with the indicated antibodies. **(G)** NDP52 and HOIPIN-8 exert minimal effects on the doxorubicin-induced intrinsic apoptotic pathway. Cells were treated with 25 μM doxorubicin and analyzed as in **(E)**. **(H)** HeLa cells were treated with 25 μM doxorubicin, 20 μM ZVAD, and/or HOIPIN-8 as indicated, and cell viability was analyzed by a Calcein-AM assay. **(A–C, H)** Data are shown as Means ± SD (*n* = 3) by Huber-White Sandwich estimators for variance-covariance structures corrected with Bonferroni method. *P < 0.05, ****P < 0.0001, NS, not significant.

TNF-α stimulation induced not only NF-κB activation but also cell death, when the expression of NF-κB targets was prohibited in the presence of cycloheximide (CHX), a protein synthesis inhibitor (47, 48). To investigate the role of NDP52 in the regulation of cell death, we treated parental and *NDP52^-/–^*-HeLa cells with TNF-α+CHX, and found that the number of trypan blue-positive dead cells was increased among *NDP52^-/-^* cells, as compared to the parental HeLa cells (**Figure 5A**). Since the pan-caspase inhibitor ZVAD suppressed the enhanced cell death, it seemed to be mediated by the apoptosis pathway. The increased TNF-α+CHX-induced cell death was detected in *NDP52*-silenced HeLa cells, as compared to the control siRNA-transfected cells (**Figure 5B**). The ATP-based cell viability assay also indicated the enhanced cell death in *NDP52^-/-^* cells after TNF-α+CHX or TNF-α+Smac mimetic BV6 treatment (**Figure 5C**). Furthermore, a real time cell analysis revealed that *NDP52^-/–^*-HeLa cells died more rapidly than the parental cells after TNF-α+CHX-treatment, and cell death was accelerated in the presence of HOIPIN-8 (**Figure 5D**). Indeed, the cleavages of PARP, caspase 8, and caspase 3, which are hallmarks of apoptosis, were increased in *NDP52^-/–^*-HeLa cells and dose-dependently enhanced by HOIPIN-8 or HOIPIN-1 (**Figure 5E**, **Supplementary Figure 6A, B**). During the course of TNF-α-induced cell death, TNFR complex II, composed of RIP1, caspase 8, and Fas-associated death domain protein (FADD), plays a crucial role to activate the apoptosis pathway (49). The anti-FADD immunoprecipitation indicated that TNF-α+CHX-induced TNFR complex II formation was upregulated in *NDP52^-/-^* cells as compared to the parental cells, and was further increased in the presence of HOIPIN-8 (**Figure 5F**). These data indicated that NDP52 physiologically functions as an anti-apoptotic factor in the TNF-α-induced apoptotic pathway.

Doxorubicin, a genotoxic agent, activates the Bcl-2/Bax-mediated intrinsic apoptosis pathway (50). In contrast to the TNF-α+CHX treatment, the cleavages of PARP, Bid, and caspases 9, 8, and 3 were similar between the doxorubicin-treated parental and *NDP52^-/–^*-HeLa cells (**Figure 5G**). In the presence of HOIPIN-8, the doxorubicin-induced cleavages of Bid, and caspases 8 and 3, but not PARP and caspase 9, were suppressed in parental and *NDP52^-/–^*-HeLa cells. However, cell viability and toxicity assessments by CellTiter-Glo, Calcein-AM, and lactate dehydrogenase analyses did not reveal a significant anti-apoptotic effect of HOIPIN-8 in doxorubicin-treated cells (**Figures 5C, H**, **Supplementary Figure 6C**). These results suggested that, in contrast to the TNF-α-induced apoptotic pathway, the intrinsic apoptotic pathway is not affected by the genetic ablation of *NDP52* and/or the inhibition of LUBAC activity.

### Effect of HOIPINs on NDP52-Mediated Xenophagy

To evaluate the importance of the crosstalk between NDP52 and LUBAC, we next investigated the effects of HOIPIN-8 on the xenophagy triggered by *Salmonella typhimurium* infection in HeLa cells. At 1 h after infection, LC3-positive membranes were recruited to *Salmonella* foci, and most of them were also positive for linear ubiquitin (**Supplementary Figures 7A, 8**) in accordance with previous reports (24, 25). In contrast, the majority of the invaded *Salmonella* cells were devoid of LC3, and the colocalization between LC3 and linear ubiquitin, which were shown by *Pearson’s* and *Manders’* correlation coefficients and mean fluorescence intensities, was significantly diminished in HOIPIN-8-treated cells (**Supplementary Figures 7B, C, 8**). At 8 h after infection, *Salmonella* escaped from the clearance system of the host cells and started to explosively proliferate in the cytoplasm, even in the non-treated cells. At this time point, the bacterial cells were covered with a trace amount of linear ubiquitin, but the LC3 positive membranes were not well recruited to the expanding bacterial foci, in both the non-treated and HOIPIN-8-treated cells (**Supplementary Figures 7D, E, 8**). The colony forming assay indicated that the entry into HeLa cells was not affected by HOIPIN-8, whereas the *Salmonella* expansion was facilitated by the suppression of LUBAC (**Supplementary Figure 7F**). Thus, the compromised elimination of bacteria in HOIPIN-8-treated cells can be attributed to the defects in the initial responses against *Salmonella*, including the linear ubiquitination and LC3 recruitment.

**Figure 6:**
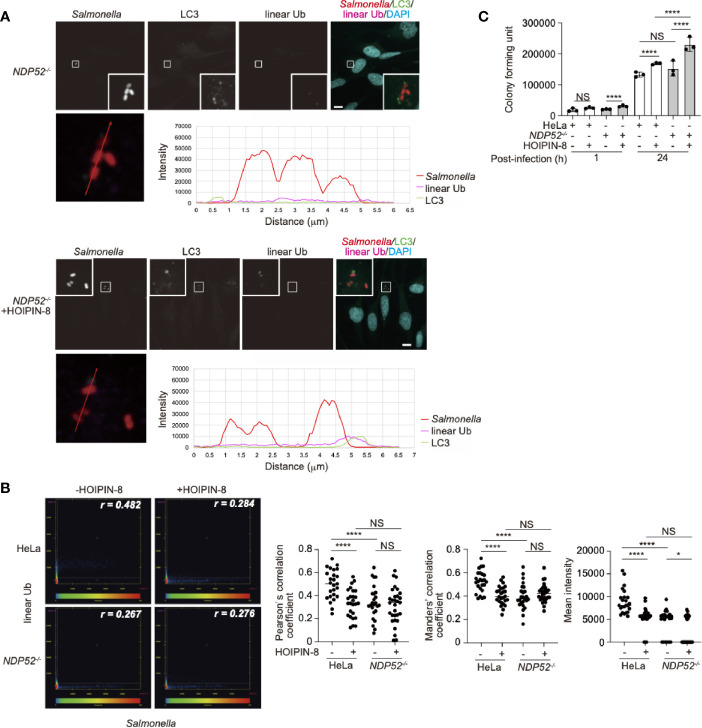
Effect of LUBAC inhibitor on xenophagosome formation in *NDP52* ^-/–^-HeLa cells. **(A)** Colocalization of linear ubiquitin with *Salmonella* xenophagosomes. *NDP52* ^-/–^-HeLa cells were infected with mCherry-labeled *Salmonella*. The recruitments of endogenous LC3 and linear ubiquitin chains were visualized by immunofluorescence analyses. Representative confocal images of each condition are shown. *Insets*: Enlarged images of boxed regions with *Salmonella* foci. Lower panels: Intensity profile plot of *Salmonella*, linear ubiquitin, and LC3 signals on the line across *Salmonella* foci shown in the left panel. *Bars*: 10 μm. **(B)** Colocalization analysis of *Salmonella* foci and linear ubiquitin. Representative dot plots from *Salmonella*-infected wild type and *NDP52* ^-/–^-HeLa cells with or without HOIPIN-8 treatment are shown. *Pearson’s* correlation coefficient (*r*) is indicated in the upper right corner of each panel. Scatter plots of *Pearson’s* and *Manders’* correlation coefficients, and mean intensities calculated from multiple images of *Salmonella* foci of each group are shown on the right side. Data are shown by scatter plots using HeLa without HOIPIN-8, (*n* = 25); HeLa with HOIPIN-8, (*n* = 28); *NDP52* ^-/-^ without HOIPIN-8, (*n* = 25); and *NDP52* ^-/-^ with HOIPIN-8, (*n* = 30). **(C)** Colony formation assay of cells infected with *Salmonella*. Parental and *NDP52^-/–^*-HeLa cells were infected with *Salmonella* at a MOI of 100 in triplicate, and cultured in the absence or presence of 30 μM HOIPIN-8. The invaded intracellular bacteria were grown on LB agar plates in quadruplicate and their colonies were counted at the indicated timepoints. Data are shown as Means ± SD (n = 3). **(B, C)** Data was analyzed by Huber-White Sandwich estimators for variance-covariance structures corrected with Bonferroni method. *P < 0.05, ****P < 0.0001, NS, not significant.

We next examined whether NDP52 affects the linear ubiquitination of the invaded *Salmonella*. *NDP52^-/–^*-HeLa cells were infected with *Salmonella* as described previously (29), and the colocalization of linear ubiquitin and *Salmonella* foci in either the absence or presence of HOIPIN-8 was evaluated and compared with that in parental HeLa cells. Interestingly, the linear ubiquitination of *Salmonella*, which was observed in parental HeLa cells, was profoundly suppressed in *NDP52^-/-^* cells (**Figure 6A**, **Supplementary Figure 9**). Quantitative imaging analyses of *Salmonella* and linear ubiquitin colocalization revealed that NDP52 plays an important role in the linear ubiquitination of invaded *Salmonella* (**Figure 6B**). We also tested the effects of HOIPIN-8 on *Salmonella* elimination in *NDP52^-/–^*-HeLa cells. In the *NDP52*-deficient background, no additional effects of LUBAC inhibition on the reduction of colocalization or linear ubiquitination were found (**Figure 6B**), but the HOIPIN-8 treatment strikingly exacerbated *Salmonell*a elimination in *NDP52^-/–^*-HeLa cells, while the NDP52 defect showed minimal adverse effects in non-treated cells (**Figure 6C**). These results suggested that NDP52 substantially contributes to the elimination of invading bacteria in collaboration with LUBAC.

We finally investigated the effect of a LUBAC inhibitor on the NDP52 localization in *Salmonella*-infected cells. At 1 h after infection, NDP52 was selectively accumulated in *Salmonella*-encapsulating xenophagosomes (**Supplementary Figures 10A, 11**). As expected, either the inhibition of LUBAC or the genetic loss of *NDP52*, or both caused poor xenophagosome formation (**Supplementary Figures 10B-D, 11**), while their effects on the colocalization correlation between *Salmonella* and LC3 were limited (**Supplementary Figure 10E**). In contrast to the reduction in LC3-positive xenophagosome formation, the NDP52 recruitment to *Salmonella* was not affected by the HOIPIN-8 treatment (**Supplementary Figure 10F**).

These xenophagy analyses indicated that LUBAC plays an important role in xenophagosome formation, and thus the suppression of LUBAC attenuates the clearance of invaded bacteria. In addition, the genetic deletion of *NDP52* also curtailed both the linear ubiquitination of *Salmonella* and LC3 recruitment, indicating that the collaboration between LUBAC and NDP52 takes a part in xenophagy. In summary, the crosstalk between NDP52 and LUBAC contributes to several cellular responses, including NF-κB-mediated inflammation, apoptosis, and xenophagy regulation.

## Discussion

**Figure 7:**
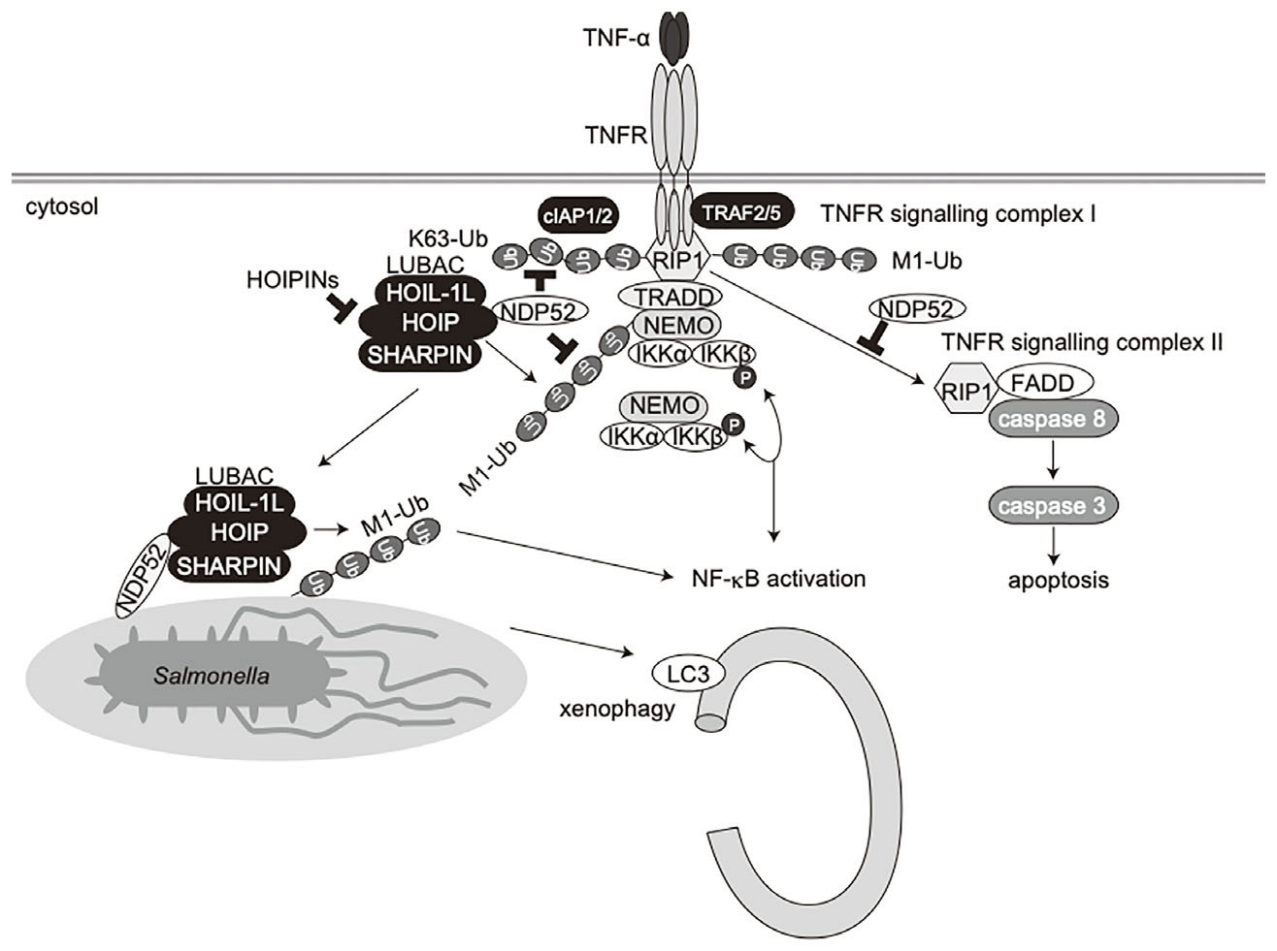
Schematic model of innate immune responses, apoptosis, and xenophagy by crosstalk between NDP52 and LUBAC.

NDP52 is a multifunctional regulatory protein that predominantly works in selective autophagy (17, 18). Although linear (de)ubiquitination is involved in the NDP52-mediated xenophagy of invading *Salmonella* (24, 25), little is known about the crosstalk between NDP52 and LUBAC in innate immune responses. In terms of the regulation of the NF-κB signaling pathway, NDP52 is reportedly involved in the TNF-α-induced NF-κB signal transduction network (41), and only slightly inhibited the TRAF6-mediated NF-κB activation (51). Furthermore, NDP52 suppressed the lipopolysaccharide-induced NF-κB and IRF3 activation pathways by mediating the autophagic degradations of Toll/interleukin-1 receptor homology domain-containing adaptor inducing interferon (TRIF) and TRAF6 (52). In this study, we showed that NDP52 down-regulates the canonical NF-κB pathway through ubiquitin-binding *via* the C-terminal UBZ domain, which is centered on a crucial Asp439 residue (**Figure 1**). The V248A variant of NDP52 is reportedly associated with Crohn’s disease, and the variant affects Toll-like receptor-mediated NF-κB signaling through the ubiquitin-binding at this region. However, V248A of NDP52 showed no effect on NF-κB activation, suggesting that it may affect other cellular functions, including selective autophagy. NDP52 reportedly associates with NF-κB signaling factors, such as the IKK complex (39, 40), TRAFs (35, 40, 42, 43), A20 (41), c-IAP2 (38), and HOIL-1L (35), and we identified HOIP as the primary NDP52-binding subunit in LUBAC (**Figure 2**). The ubiquitin-binding defective mutants of NDP52 may have a positive effect on the NF-κB activity through their different binding affinities for NF-κB signaling factors, such as by the substantial binding with IKKα/β. Importantly, NDP52 is a component of TNFR signaling complex I (**Figure 3**). At present, K11-, K63-, and M1-ubiquitin chains, which are produced by the E3s of c-IAP-1/2, TRAF2/5, and LUBAC, are known to be involved in TNFR signaling complex I (45, 53, 54). Moreover, the linear ubiquitination of NEMO functions as a scaffold to recruit multiple IKK molecules through the UBAN domain of NEMO, and induces the *trans*-activation of canonical IKK (44, 55). The ubiquitin-binding of NDP52 through the UBZ domain showed its broad specificity in ubiquitin-linkages (32), although it also appeared to have affinities toward atypical linkages, such as K27-, K29-, K63-, and M1-chains (**Figure 1**). Therefore, NDP52 seems to be recruited to TNFR by binding with a wide variety of ubiquitin chains, including the M1 chain, and thus regulates the canonical NF-κB activation pathway (**Figure 7**). Indeed, the recruitment of NDP52 to TNFR complex I was suppressed in *HOIP*-deficient cells (**Supplementary Figure 4**).

In addition to its role in NF-κB signaling, NDP52 partly regulates the type I IFN production pathway, and a LUBAC inhibitor cancelled the enhanced antiviral signaling in *NDP*52-deficient cells (**Figure 4**). Since NDP52 reportedly associates with several antiviral signaling factors, such as TBK1 (36), TBKBP1 (36), MAVS (37), and IKKϵ (21), it seems to function as a crucial regulator in innate immune responses.

Furthermore, we showed that the TNF-α-induced apoptotic pathway, but not doxorubicin-mediated intrinsic apoptosis, was enhanced in *NDP52^-/–^*-HeLa cells as compared to parental HeLa cells (**Figure 5**). When TNFR signaling complex I fails to activate NF-κB, cells activate the apoptotic pathway with the formation of a second complex, complex II, composed of RIP1, FADD, and caspase 8 (49). Indeed, we identified the facilitated complex II formation in *NDP52*-deficient cells upon TNF-α+CHX treatment (**Figure 5**). In the course of complex II formation, RIP1 deubiquitination by CYLD, a deubiquitinase that cleaves linear- and K63-ubiquitin chains, plays a crucial role (56, 57). Therefore, NDP52 may prevent the CYLD-mediated deubiquitination of RIP1, resulting in the suppression of the TNF-α-induced apoptotic pathway (**Figure 7**). Since HOIPIN-8 inhibits the LUBAC-mediated linear ubiquitination of RIP1 upon TNF-α stimulation, it also seems to facilitate the complex II formation.

In addition to NDP52, other multiple autophagy receptors, including OPTN, p62 and TAX1BP1, are also involved in xenophagy, although NDP52 seems to play a major role in *Salmonella* elimination (21–23, 58). Moreover, LUBAC and OTULIN are crucial regulators of xenophagy (24, 25), and LUBAC linearly ubiquitinates proteins in the inner membrane of invading *Salmonella*, and preferentially recruits linear ubiquitin-specific UBAN domain-containing OPTN and NEMO, which facilitate xenophagy and NF-κB activation, respectively (24). LUBAC and OTULIN are reportedly regulates the initiation of autophagy by the linear (de)ubiquitination of ATG13 (26), suggesting the crosstalk between linear ubiquitination and selective autophagy. In this study, we showed that *Salmonella*, LC3, and linear ubiquitin are colocalized 1 h after infection, and HOIPIN-8 treatment suppressed the recruitment of linear ubiquitin and LC3 to the bacteria, thereby allowing increased colony formation (**Supplementary Figure 7**). On the other hand, NDP52 recruitment was scarcely disturbed by HOIPIN-8 treatment (**Supplementary Figure 10**), consistent with previous findings that OPTN recruitment to *Salmonella* was abrogated in *HOIP*-deficient cells, in contrast to NDP52 (24). Intriguingly, the ablation of *NDP52* significantly reduced the linear ubiquitination of *Salmonella* (**Figure 6**), suggesting that NDP52 is not an inhibitor of LUBAC in xenophagy progression, but is required for the effective linear ubiquitination of invading bacteria and xenophagosome formation. Thus, apart from its adaptor function, the NDP52 recruited to bacterial foci might work cooperatively with LUBAC in forming xenophagosomes and killing bacteria (**Figure 7**). Since the *NDP52*-deficiency alone seemed to have a minor effect, OPTN may play a dominant role in xenophagy, as reported previously (24). Further analyses of xenophagy receptors, such as the effects of the combined deletion of *NDP52* and *OPTN*, will be necessary.

Collectively, we have shown that NDP52 functions in innate immune responses, such as the NF-κB and type I IFN antiviral signaling pathways, apoptosis, and selective autophagy such as xenophagy (**Figure 7**). Therefore, the crosstalk between NDP52 and LUBAC may represent an attractive therapeutic target to develop anti-inflammatory agents.

## Data Availability Statement

The original contributions presented in the study are included in the article/**Supplementary Material**. Further inquiries can be directed to the corresponding author.

## Ethics Statement

The protocols were approved by the Safety Committee for Recombinant DNA Experiments and the Safety Committee for Bio-Safety Level 2 (BSL-2) Experiments of Osaka City University.

## Author Contributions

HM, DO, and ST performed cell biological experiments, DK and AS performed statistical analyses, and FT coordinated the study. All authors wrote and commented on the manuscript, and discussed the results. All authors contributed to the article and approved the submitted version.

## Funding

This work was partly supported by MEXT/JSPS KAKENHI grants (Nos. JP16H06276 (AdAMS), JP16H06575, JP18H02619, and JP19K22541 to FT, JP18K06967, JP19H05296, and JP20H05337 to DO, and JP18K06937 to ST), a grant from the Takeda Science Foundation (to FT and ST), and a Grant for Research Program on Hepatitis from the Japan Agency for Medical Research and Development (AMED – 19fk0210050h0001 to FT).

## Conflict of Interest

The authors declare that the research was conducted in the absence of any commercial or financial relationships that could be construed as a potential conflict of interest.
