# Supplementary material for: Crosstalk Between NDP52 and LUBAC in Innate Immune Responses, Cell Death, and Xenophagy
Source: Front Immunol. 2021 Mar 19;12:635475. doi: 10.3389/fimmu.2021.635475 (PMC8017197; doi:10.3389/fimmu.2021.635475)
Supplement: Supplementary file 1 [file DataSheet_1.pdf]

Supplementary Material

Crosstalk between NDP52 and LUBAC in innate immune responses, cell death, and xenophagy

Hirohisa Miyashita, Daisuke Oikawa, Seigo Terawaki, Daijiro Kabata, Ayumi Shintani, and Fuminori Tokunaga

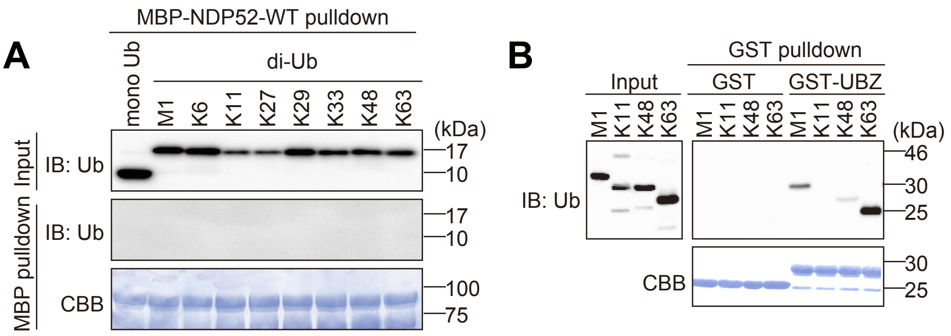

Supplementary Figure 1. *In vitro* ubiquitin binding assay of NDP52. (**A**) Mono- and eight kinds of diubiquitins were pulled down by MBP-NDP52-WT (a.a. 1-446). The input and precipitates were then immunoblotted with an anti-ubiquitin antibody. MBP-fused proteins were detected with Coomassie Brilliant Blue (CBB) staining. (**B**) A similar analysis as in Figure 1B was performed by using GST or GST-fused UBZ domain (a.a. 394-446), and M1-, K11-, K48-, or K63-tetraubiquitins.

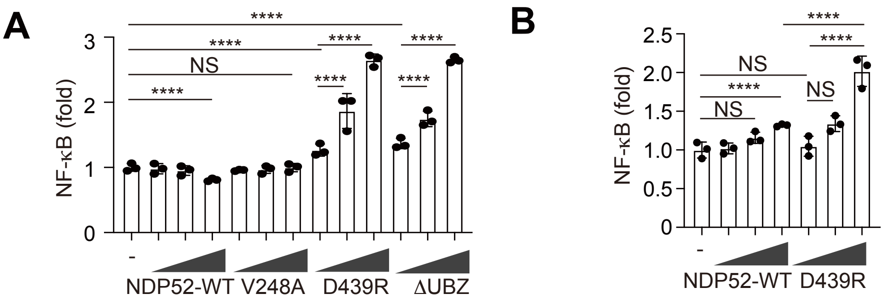

Supplementary Figure 2. Effect of NDP52 on the basal NF-κB activity. (**A**) The NF-κB luciferase reporter was co-transfected with increasing amounts (0.025, 0.075, and 0.25 μg/well) of NDP-52-WT, V248A, D439R, and ΔUBZ-mutants in HEK293T cells. (**B**) A similar analysis as in (**A**) was performed using the NDP-52-WT and D439R mutant in A549 cells. Data are shown as Means ±SD (*n* = 3) and were analyzed by Huber-White Sandwich estimators for variance-covariance structures corrected with Bonferroni method. ****: $P<0.0001$, NS: not significant.

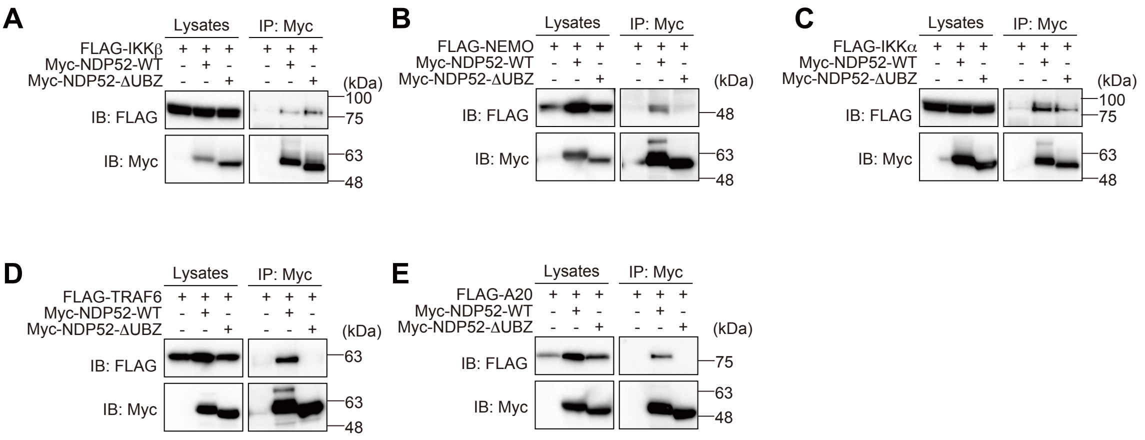

Supplementary Figure 3. NDP52 binds multiple NF-κB signaling factors. FLAG-tagged NF-κB signaling factors, such as IKKβ (**A**), NEMO (**B**), IKKα (**C**), TRAF6 (**D**), and A20 (**E**), were co-transfected with the Myc-NDP52-WT or ΔUBZ mutant in HEK293T cells. The cell lysates and Myc-immunoprecipitates were then immunoblotted with the indicated antibodies.

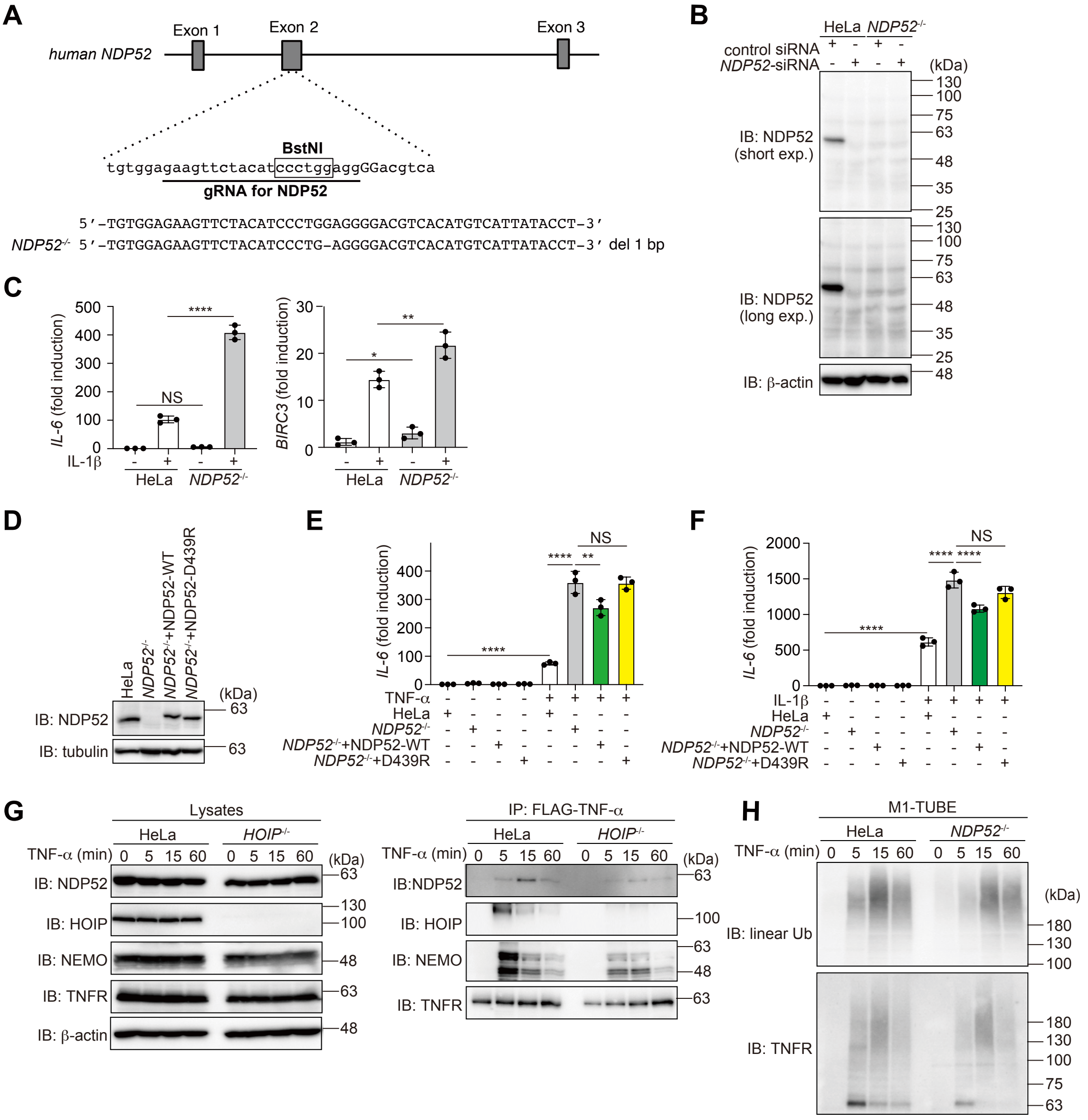

Supplementary Figure 4. Construction and characterization of *NDP52*-deficient HeLa cells. (**A**) Scheme for *NDP52*-KO targeting. The gRNA was employed to target exon 2 of the *NDP52* gene in HeLa cells. The nucleotide sequence of the constructed *NDP52*$^{-/-}$-HeLa cells is shown. (**B**) Evaluation of *NDP52*-deficiency. *NDP52* was knocked down by siRNA in parental and *NDP52*-deficient HeLa cells, and cell lysates were immunoblotted with the depicted antibodies. (**C**) Enhanced expression of NF-κB target genes in IL-1β-treated *NDP52*$^{-/-}$-HeLa cells. A similar qPCR analysis as in Figure 3B was performed after 1 h stimulation with 1 ng/ml IL-1β. (**D**) Restoration of NDP52-WT and NDP52-D439R into *NDP52*$^{-/-}$ HeLa cells. *NDP52*$^{-/-}$ HeLa cells stably expressing FLAG-NDP52-WT or FLAG-NDP52-D439R were immunoblotted with the indicated antibodies.

(**E**, **F**) Restoration of NDP52-WT, but not D439R, suppressed NF-κB activation. Parental, *NDP52*$^{-/-}$, NDP52-WT- and FD439R-restored cells were stimulated with or without 10 ng/ml TNF-α (**E**) or 1 ng/ml IL-1β (**F**) for 1 h, and the expression of *IL-6* was assessed by qPCR. (**G**) Reduced recruitment of NDP52 to TNFR complex I in *HOIP*$^{-/-}$-HeLa cells. Parental and *HOIP*$^{-/-}$-HeLa cells were analyzed as in Figure 3D. (**H**) Similar linear ubiquitination in *NDP52*$^{-/-}$ cells to parental HeLa cells. Parental and NDP52$^{-/-}$-HeLa cells were stimulated with 1 μg/ml FLAG-TNF-α for the indicated periods, and cell lysates were pulled down with M1-TUBE. The precipitates were subjected to immunoblotting with the indicated antibodies. (**C**, **E**, **F**) Data are shown as Means ±SD (n = 3) and were analyzed by Huber-White Sandwich estimators for variance-covariance structures corrected with Bonferroni method. *: $P<0.05$, **: $P<0.01$, ****: $P<0.0001$, NS: not significant.

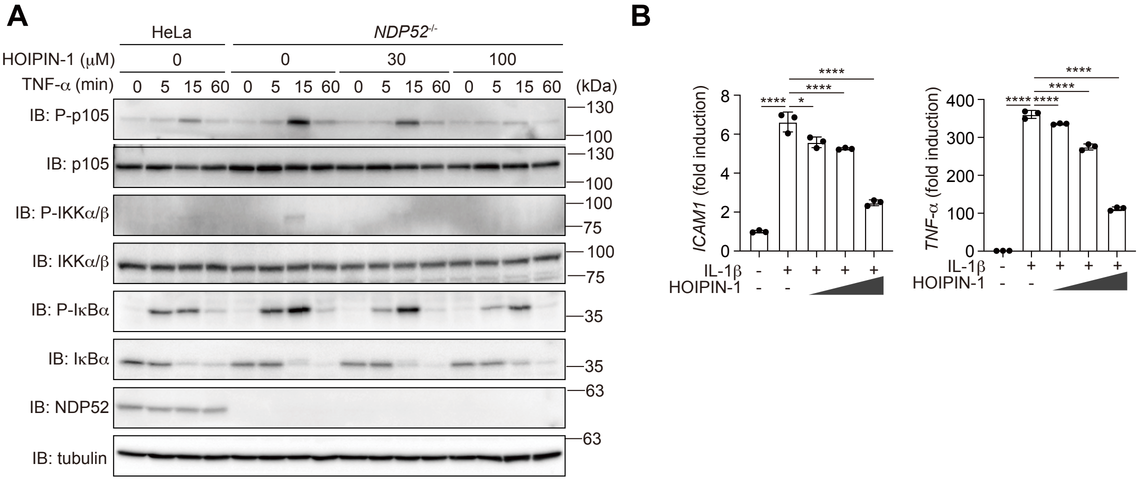

Supplementary Figure 5. HOIPIN-1 suppresses the enhanced NF-κB activation in *NDP52*$^{-/-}$ cells. (**A**) Dose-dependent suppression of the enhanced TNF-α signaling in *NDP52*$^{-/-}$-HeLa cells by HOIPIN-1. A similar analysis as in Figure 4A was performed in the presence of the indicated concentrations of HOIPIN-1. Cell lysates were immunoblotted with the depicted antibodies. (**B**) HOIPIN-1 suppresses the induction of NF-κB target genes. HeLa cells were treated with 1 ng/ml IL-1β in the absence or presence of increasing concentrations (10, 30, and 100 μM) of HOIPIN-1 for 1 h, and the mRNA levels of *ICAM1* and TNF-α were analyzed by qPCR. Data are shown as Means ±SD (n = 3) and were analyzed by Huber-White Sandwich estimators for variance-covariance structures corrected with Bonferroni method. *: P<0.05, ****: P<0.0001.

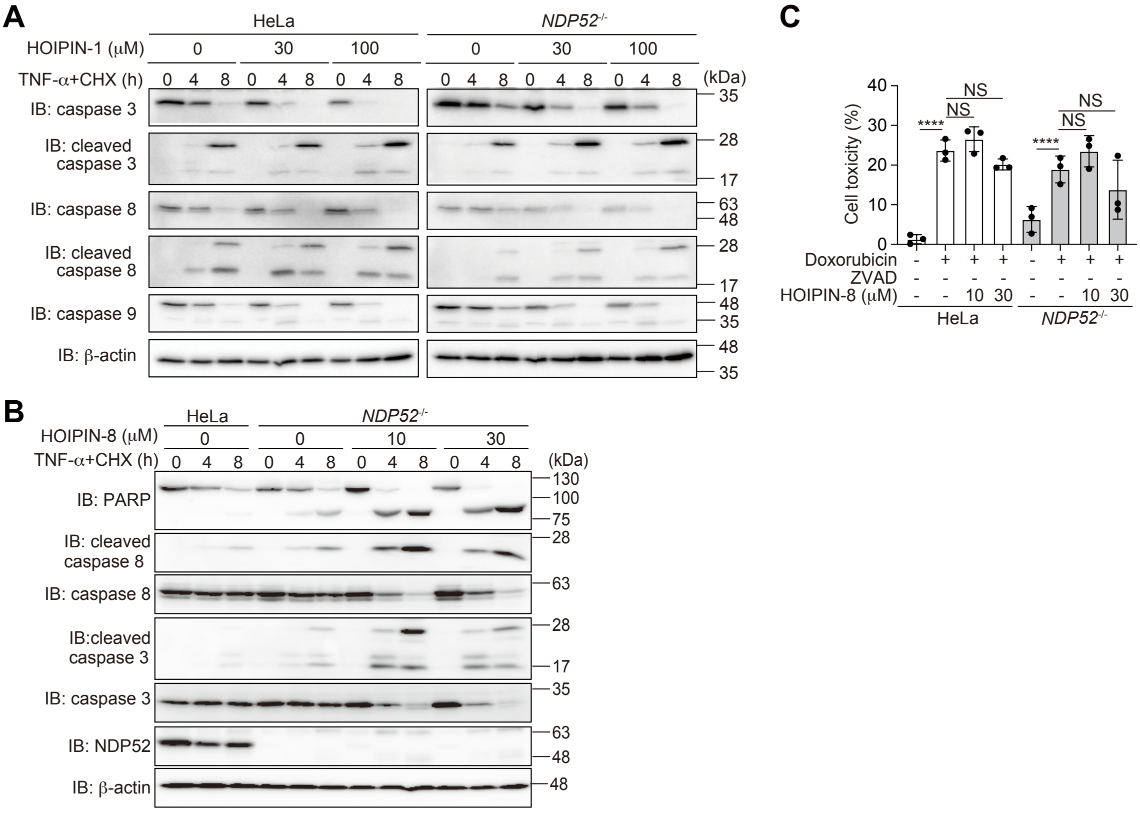

Supplementary Figure 6. Genetic ablation of *NDP52* enhances TNF-α-induced apoptosis. (**A**) Enhanced apoptosis in TNF-α+CHX- and HOIPIN-1-treated *NDP52*$^{-/-}$-HeLa cells. Parental and *NDP52*$^{-/-}$-HeLa cells were treated with 10 ng/ml TNF-α, 10 μg/ml CHX, and/or the indicated concentrations of HOIPIN-1, and cell lysates were analyzed by immunoblotting with the indicated antibodies. (**B**) A similar analysis as in (**A**) was performed, using the indicated concentrations of HOIPIN-8. (**C**) The effect of cell toxicity. Parental and *NDP52*$^{-/-}$-HeLa cells were treated with 25 μM doxorubicin, 20 μM ZVAD, and/or the indicated concentrations of HOIPIN-8 for 12 h, and cell toxicity was assessed by an LDH assay. Data are shown as Means ±SD (n = 3) and were analyzed by Huber-White Sandwich estimators for variance-covariance structures corrected with Bonferroni method. ****: P<0.0001, NS: not significant.

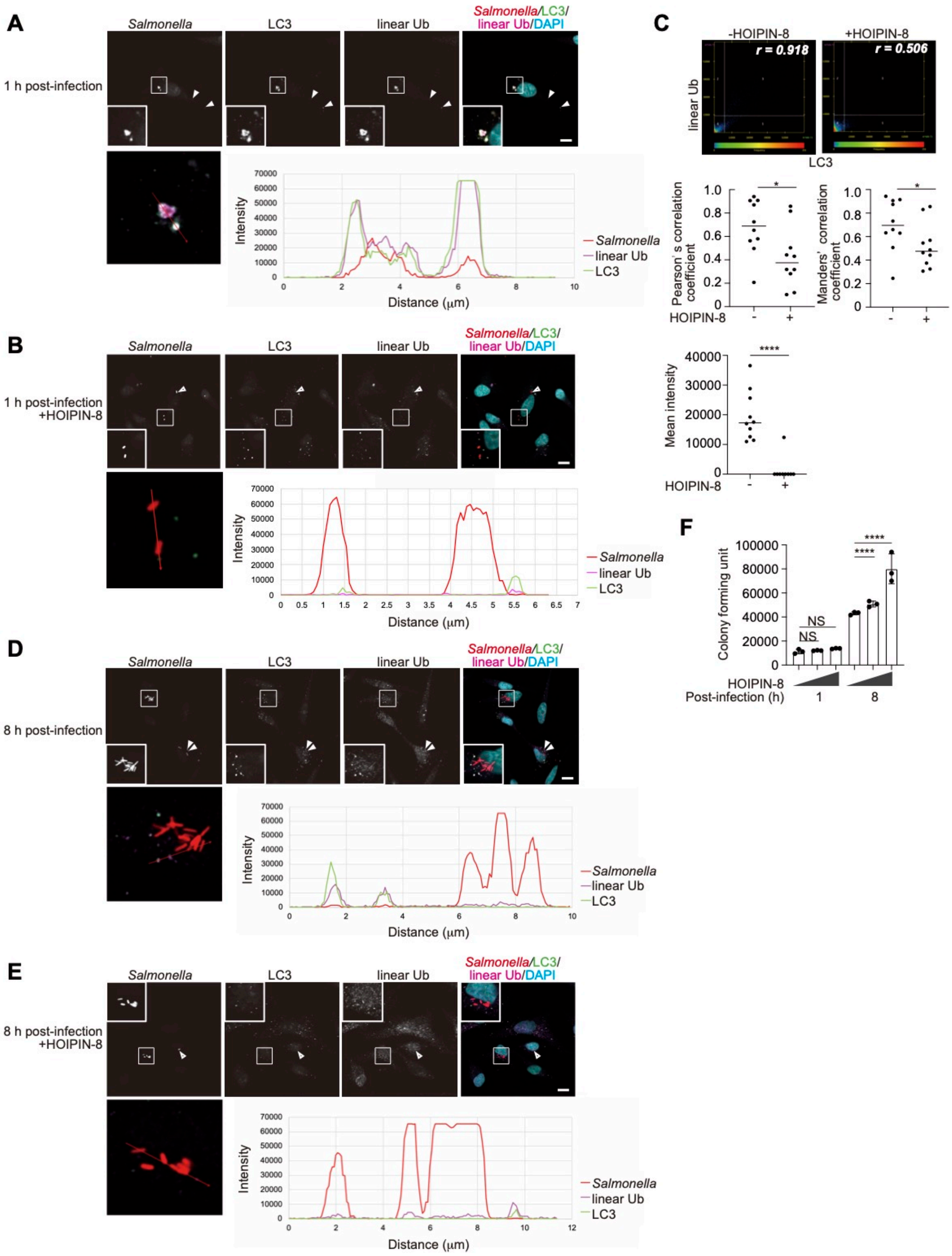

Supplementary Figure 7. LUBAC inhibitor suppresses xenophagy. (**A, B**) Inhibition of linear ubiquitination diminishes xenophagosome formation and *Salmonella* elimination. HeLa cells were infected with mCherry-labeled *Salmonella*, and then cultured for 1 h in the absence (**A**) or presence (**B**) of 30 μM HOIPIN-8. The cells were then fixed and immunofluorescence staining was performed with anti-LC3 and anti-linear ubiquitin

antibodies, and DAPI. Representative confocal images of each condition are shown. *Insets*: Enlarged images of boxed regions with *Salmonella* clusters. *Solid triangles*: LC3-positive vesicles colocalizing with linear ubiquitin. *Open triangles*: *Salmonella* foci with poor LC3 recruitment and linear ubiquitination. Lower panels: Intensity profile plots of *Salmonella*, linear ubiquitin, and LC3 signals on the line across the *Salmonella* foci shown in the left panel. *Bars*: 10 μm. (**C**) HOIPIN-8 suppresses the colocalization of LC3 and linear ubiquitin. Dot plot colocalization analyses between LC3 and linear ubiquitin around *Salmonella* foci in (**A**) and (**B**) are depicted. The *Pearson's* correlation coefficient (*r*) of each combination of two signals is shown on the top of each panel (upper). Scatter plots representing the distribution of the *Pearson's* correlation coefficients, *Manders'* correlation coefficients, and mean intensity of LC3 from randomly selected, independent images of *Salmonella* foci in each experimental setting (lower, $n = 10$). (**D, E**) HOIPIN-8 suppresses LC3 recruitment to invaded *Salmonella*. Similar analyses as in (**A**) and (**B**) were performed in the absence (**D)** or presence (**E**) of 30 μM HOIPIN-8 for 8 h. *Bars*: 10 μm. (**F**) Xenophagy is suppressed by a LUBAC inhibitor. HeLa cells were infected with *Salmonella* at a MOI of 100. The colonies formed by invading *Salmonella* were counted on LB agar plates inoculated with serially diluted cell lysates, prepared in quadruplicate at 1 h- and 8 h-post infection, in the presence of 0, 10, and 30 μM HOIPIN-8. Means ±SD ($n = 3$) (**C**, **F**) Data were analyzed by Huber-White Sandwich estimators for variance-covariance structures corrected with Bonferroni method. *: $P<0.05$, *****: $P<0.0001$, NS: not significant.

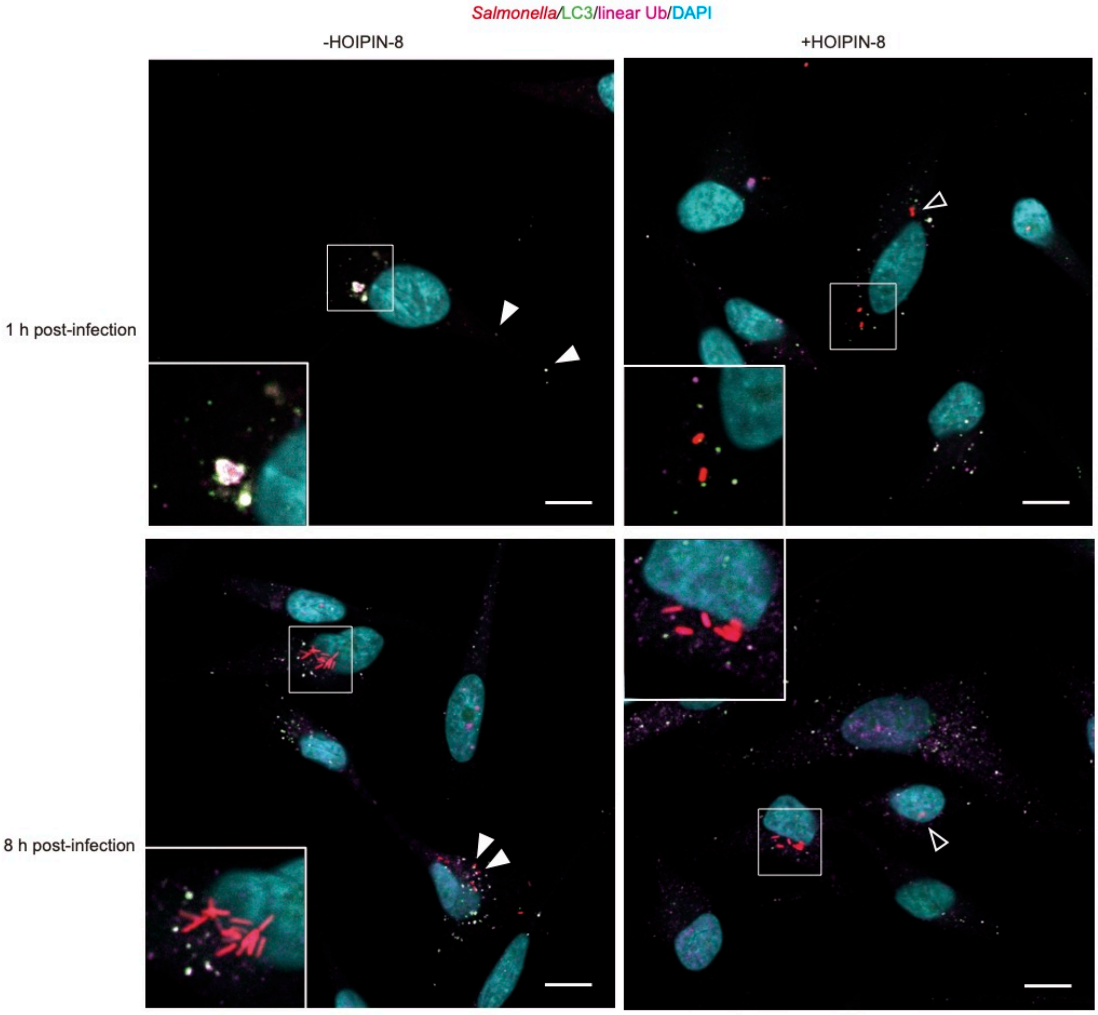

Supplementary Figure 8. Enlarged merged images shown in Supplementary Figure 7. *Bars*: 10 µm.

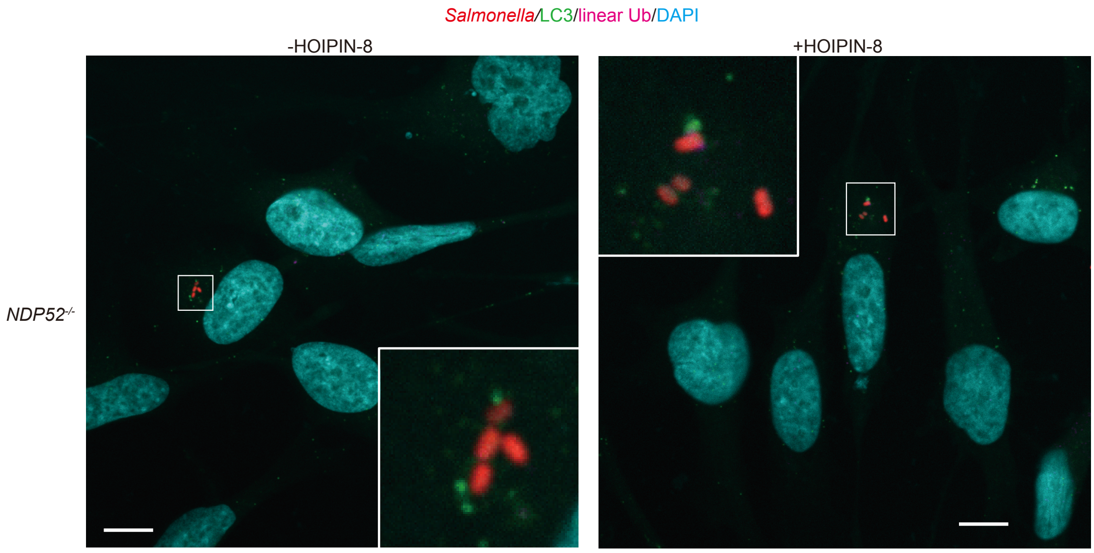

Supplementary Figure 9. Enlarged merged images shown in Figure 6. *Bars*: 10 µm.

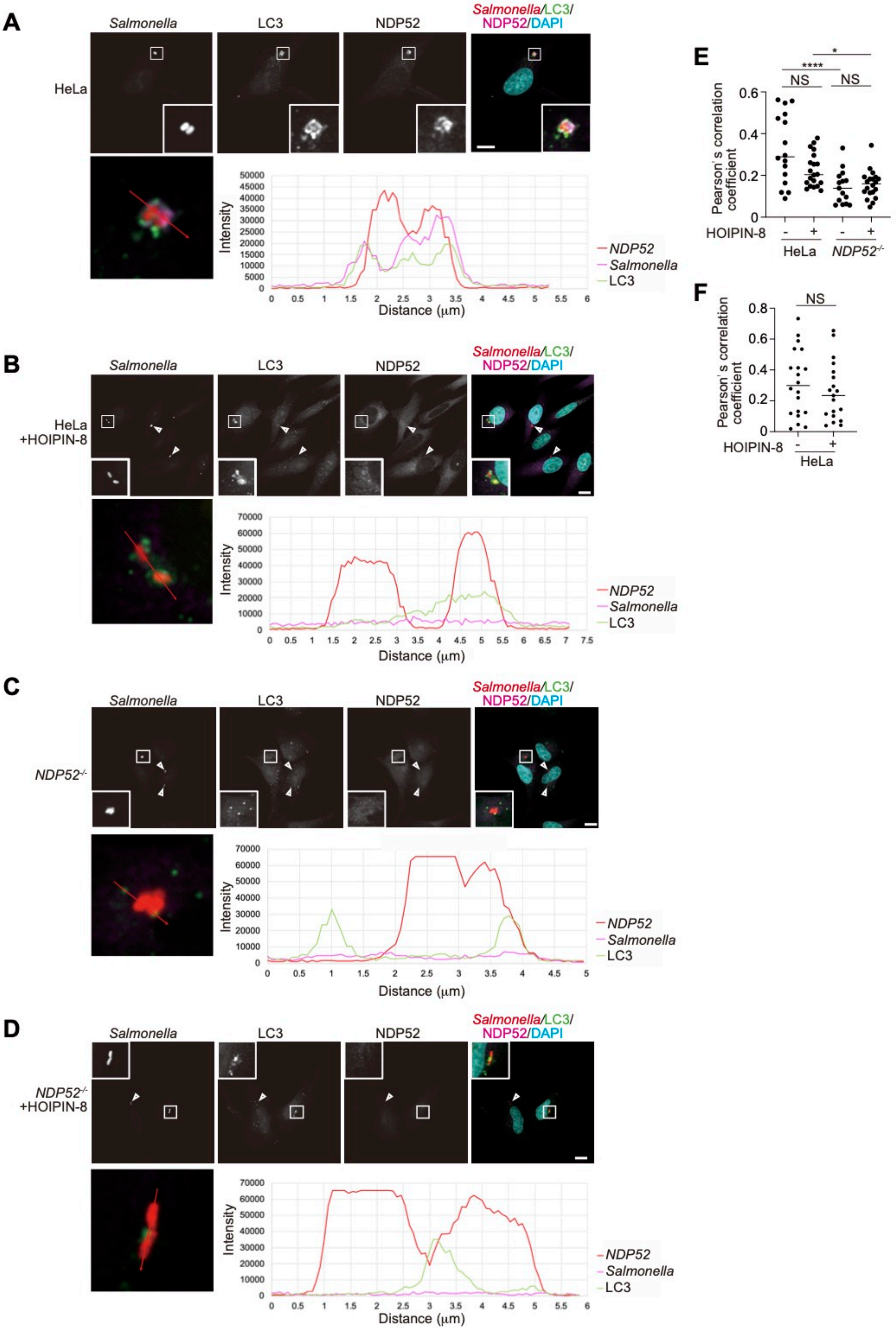
A
Salmonella
LC3
NDP52
Salmonella/LC3/NDP52/DAPI
HeLa
Intensity
Distance (μm)
NDP52
Salmonella
LC3
B
HeLa +HOIPIN-8
C
NDP52-/-
D
NDP52-/- +HOIPIN-8
E
Pearson's correlation coefficient
NS
****
*
HOIPIN-8
HeLa
NDP52-/-
F
HOIPIN-8
HeLa

Supplementary Figure 10. Effect of LUBAC inhibitor on xenophagosome formation in *NDP52*$^{-/-}$-HeLa cells. (**A-D**) Colocalization of NDP52 with *Salmonella* xenophagosomes. Parental and *NDP52*$^{-/-}$-HeLa cells were infected with mCherry-labeled *Salmonella*, cultured for 1 h in the absence or presence of 30 μM HOIPIN-8, and then immunolabeled for LC3 and NDP52 after fixation. Representative confocal images of each condition are shown. *Insets*: Enlarged images of boxed regions with *Salmonella* foci. *Open triangles*: *Salmonella* foci with poor LC3 recruitment. *Bars*: 10 μm. (**E)** Colocalization analyses of *Salmonella* and LC3. Scatter plots of *Pearson's* correlation coefficients between LC3 and *Salmonella* from multiple randomly selected *Salmonella* foci images are shown using HeLa-HOIPIN-8, (n = 16); HeLa+HOIPIN-8, (n = 20); *NDP52*$^{-/-}$-HOIPIN-8, (n = 15); and *NDP52*$^{-/-}$+HOIPIN-8, (n = 20). (**F**) NDP52 recruitment to *Salmonella* in the absence or presence of HOIPIN-8. A scatter plot of *Pearson's* correlation coefficients between NDP52 and *Salmonella* from multiple *Salmonella* foci images of WT HeLa cells is shown using HeLa-HOIPIN-8, (n = 20) and HeLa+HOIPIN-8, (n = 19). (**E**, **F**) Data were analyzed by Huber-White Sandwich estimators for variance-covariance structures corrected with Bonferroni method. *: P<0.05, *****: P<0.0001, NS: not significant.

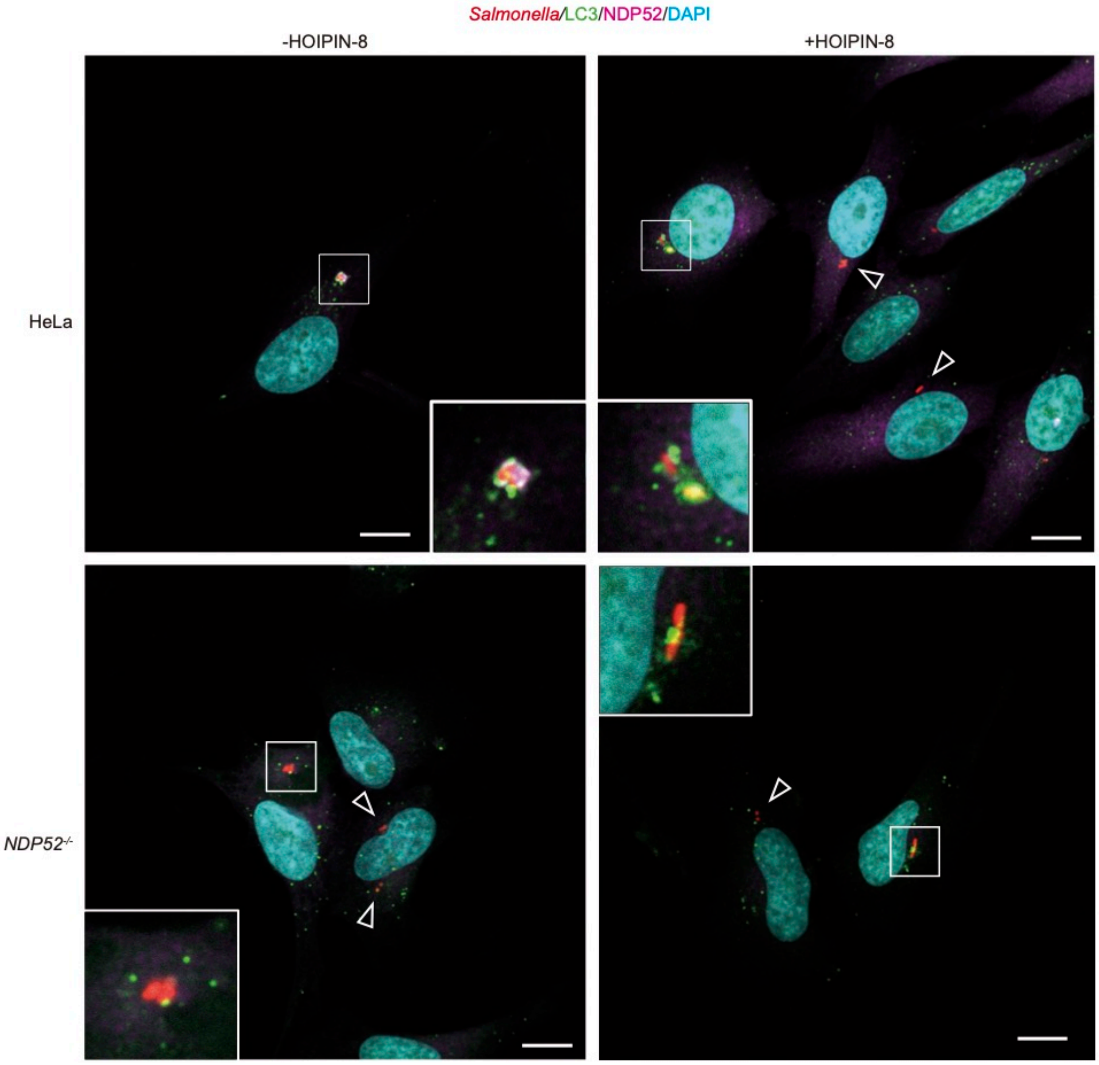

Supplementary Figure 11. Enlarged merged images shown in Supplementary Figure 10. *Bars*: 10 µm.
